# Robust Organizational Principles of Protrusive Biopolymer Networks in Migrating Living Cells

**DOI:** 10.1371/journal.pone.0014471

**Published:** 2011-01-18

**Authors:** Björn Stuhrmann, Florian Huber, Josef Käs

**Affiliations:** Institute of Soft Matter Physics, University of Leipzig, Leipzig, Germany; Max Delbrück Centre for Molecular Medicine, Germany

## Abstract

Cell migration is associated with the dynamic protrusion of a thin actin-based cytoskeletal extension at the cell front, which has been shown to consist of two different substructures, the leading lamellipodium and the subsequent lamellum. While the formation of the lamellipodium is increasingly well understood, organizational principles underlying the emergence of the lamellum are just beginning to be unraveled. We report here on a 1D mathematical model which describes the reaction-diffusion processes of a polarized actin network in steady state, and reproduces essential characteristics of the lamellipodium-lamellum system. We observe a steep gradient in filament lengths at the protruding edge, a local depolymerization maximum a few microns behind the edge, as well as a differential dominance of the network destabilizer ADF/cofilin and the stabilizer tropomyosin. We identify simple and robust organizational principles giving rise to the derived network characteristics, uncoupled from the specifics of any molecular implementation, and thus plausibly valid across cell types. An analysis of network length dependence on physico-chemical system parameters implies that to limit array treadmilling to cellular dimensions, network growth has to be truncated by mechanisms other than aging-induced depolymerization, e.g., by myosin-associated network dissociation at the transition to the cell body. Our work contributes to the analytical understanding of the cytoskeletal extension's bisection into lamellipodium and lamellum and sheds light on how cells organize their molecular machinery to achieve motility.

## Introduction

Cell motility is of vital importance for the development and maintenance of multicellular organisms. The directed crawling of animal cells is at the root of physiological processes such as wound healing, immune defense, and the remodeling and regeneration of the nervous system. Cell motility involves reorganization of the cell cytoskeleton, an intricate composite network of biopolymer filaments spanning the cell and endowing it with structure, mechanical stability, and function [1]–[3]. It is currently accepted that protrusion of the cell front is achieved by polar growth of biopolymer filaments of the protein actin against the cell membrane [4]. Modulated by a multitude of regulatory proteins, this process results in a highly dynamic, sheet-like extension [5]. At closer inspection this extension is no homogenous entity but is comprised of two spatially distinct sub-networks designated as the lamellipodium which makes up the first 1–2 µm of the cell front, and the lamellum behind [6], [7], with diverse characteristics in terms of structure, molecular composition, kinetics, and kinematics.

The molecular mechanisms by which the distinct features of the lamellipodium and the lamellum emerge are as yet poorly understood, despite the fact that experiments on cells as well as reconstituted motility systems [8], [9] have identified the essential molecular players and catalyzed a burst of theoretical modeling of different aspects of lamellipodium protrusion, reviewed, e.g., by Mogilner [10] and Carlsson et al. [11]. Few of these studies [12]–[20] extend behind the leading 1–2 µm of the cell's protruding edge, and up to now only two studies address mechanisms of lamellum generation [12], [13].

**Figure 1:**
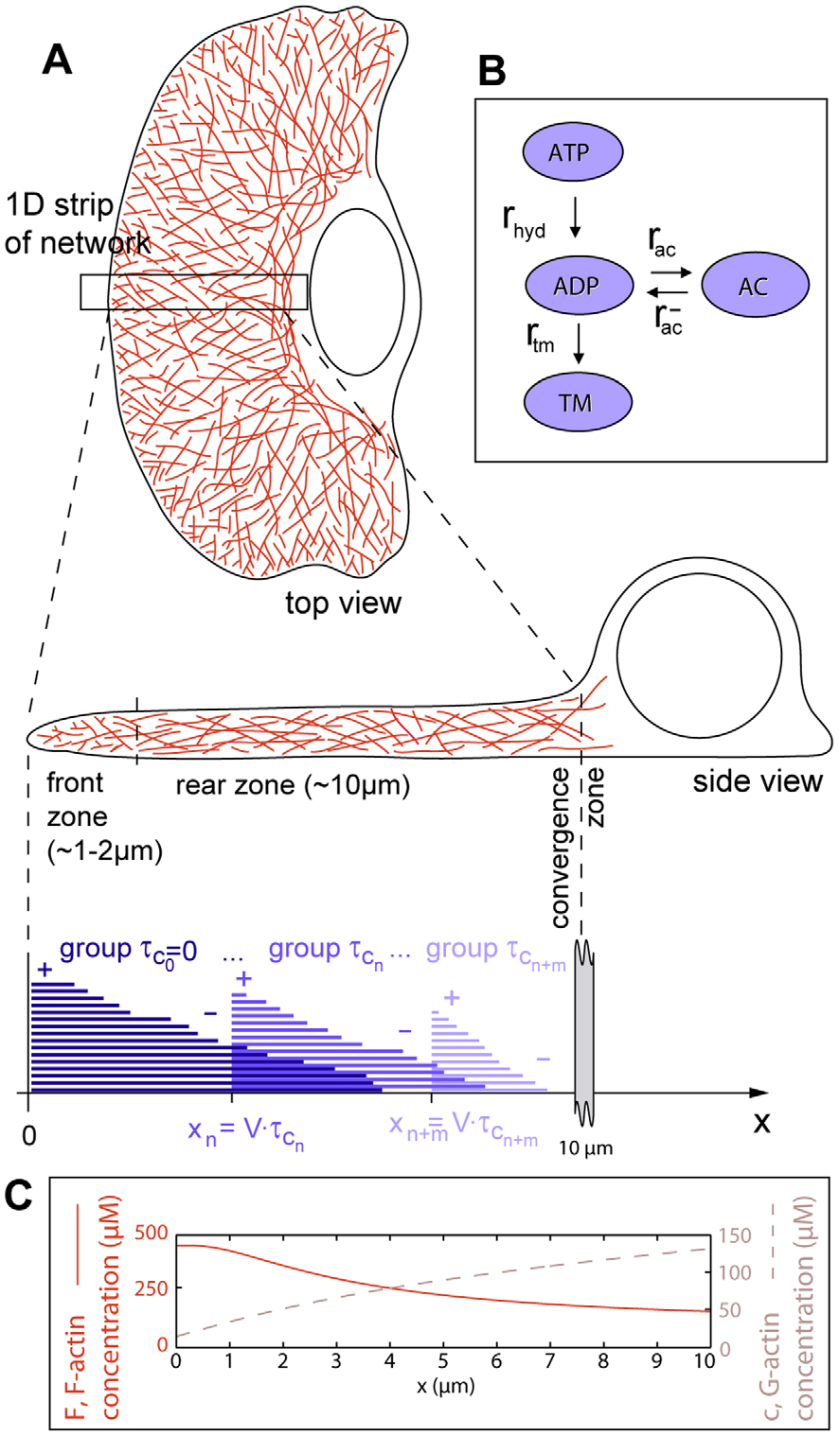
Model system. (A) Geometry of the modelled system. The scope of the one-dimensional model is a narrow strip in the center of the leading cytoskeletal extension of a keratocyte moving in the –*x* direction. Lower panel: Uncapped actin filaments (*τ_c_* = 0) grow at the cell's leading edge (*x* = 0) and depolymerize at their rear. Capped filaments move in the +*x* direction while depolymerizing and are divided into groups based on the duration *τ_c_* since they got capped. Three random representatives of the infinite number of groups are shown for clarity. The back of the system is identified with instantaneous depolymerization of transgressing filaments (convergence zone). (B) Attainable states and transformation rates for an ATP-actin subunit after its addition to a filament plus-end. ATP: ATP-F-actin (start state), ADP: ADP-F-actin, AC: ADF/cofilin-F-actin, TM: tropomyosin-F-actin. (C) Typical distributions of monomeric (G-) and filamentous (F-) actin predicted by our model.

**Figure 2:**
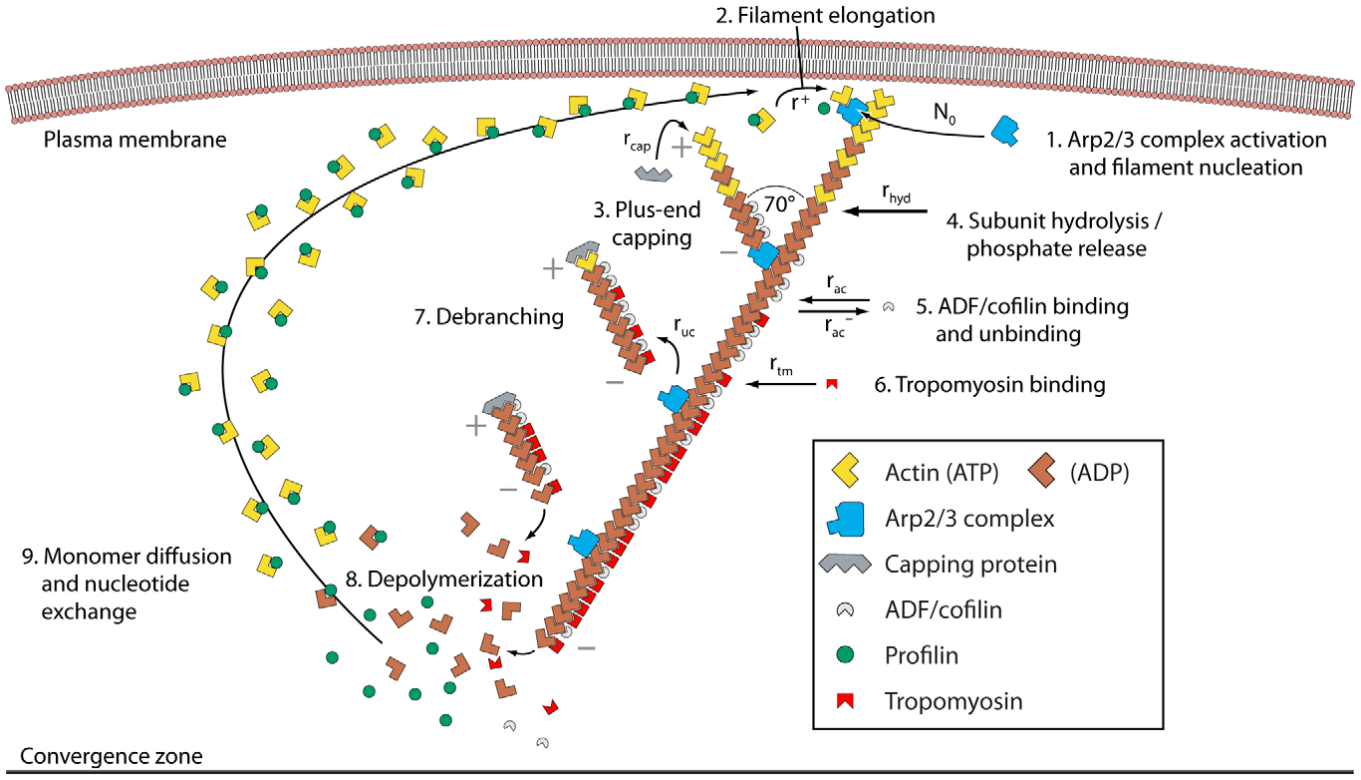
Array treadmilling providing cell front protrusion as modeled in the reported work. Actin filaments are nucleated by the Arp2/3 protein complex (activated by membrane-associated WASp/Scar proteins) as branches on existing filaments close to the plasma membrane (1) and push the membrane forward by the addition of actin monomers to their plus-ends (2). Filament growth ceases after stochastic capping of filament plus-ends (3). Filaments age by hydrolysis of ATP-nucleotide bound to each subunit and subsequent phosphate release, turning ATP-F-actin into ADP-F-actin subunits (4). ADF/cofilin (5) and tropomyosin (6) compete for binding to ADP-F-actin subunits. While tropomyosin binding is irreversible, ADF/cofilin can unbind to account for its deactivation by, e.g., LIM kinases. After debranching (detachment of the minus-end from the Arp2/3 complex, (7)), filaments depolymerize from their minus-ends (8) with a rate which is modulated by the presence of ADF/cofilin or tropomyosin on the terminal actin subunit, accounting for the regulatory effects of these proteins. Filamentous actin extending up to the contractile “convergence zone” in the back rigorously depolymerizes (process not shown). Actin monomers diffuse to the leading edge (9), whereby profilin restores their polymerization competence by catalyzing ADP-ATP nucleotide exchange.

We present here an analytical description of the essential reaction-diffusion processes in the entire leading extension of migrating cells (Figure 1). This work is based on the model assumptions of our previously published Monte Carlo simulation [12]. Arp2/3 induced nucleation, polymerization, transport, and decay of filamentous actin as well as diffusion of monomeric actin (Figure 2) are presented as a closed set of analytical equations describing the system steady state. We do not include differential substrate adhesion of the network, and hence show which of the experimentally observed network properties are independent of local adhesion site formation. This is complementary to recent studies which reproduce the kinematics of the interface between the lamellipodium and the lamellum by modeling local friction induced network stresses and concomitant network dissolution [13].

We reproduce kinetic, molecular, and structural characteristics as they are commonly observed in the lamellipodium and the lamellum of cells, in excellent agreement with our simulation data [12]. By nature, analytical descriptions of a problem offer a new quality of understanding compared to simulations. In formulating the equations of our model we were able to identify the organizational principles underlying network feature formation more precisely than in our previous work. Importantly, we now untie these organizational principles from the specifics of a cell type's distinct molecular inventory, and with this generalize our understanding of cell front organization.

Furthermore, the presented analytical work expands the results of our previous simulation by a quantitative elucidation of the extension and kinematics of a treadmilling network as a function of biochemical parameters. This is possible due to a decrease of computation time by one to two orders of magnitude. We find that mechanisms other than aging-induced network depolymerization are necessary to explain the short extent of lamellum networks observed in cells.

### Characteristics defining the lamellipodium and the lamellum

The lamellipodium makes up the frontal ∼2 µm of the cytoskeletal extension of migrating cells and is characterized by short filaments at its front which are highly branched by the Arp2/3 protein complex [21], [22]. The lamellum extends from the lamellipodium up to the convergence zone, which marks the transition to the cell body, and is characterized by an absence of Arp2/3 complex and the predominance of long, unbranched filaments [4], [22]. While the lamellipodium shows a high level of actin bound ADF/cofilin protein that destabilizes actin filaments, the lamellum is dominated by the actin stabilizer tropomyosin [7], [23], [24]. Speckle microscopy techniques have revealed actin polymerization dynamics within the cytoskeletal extension [24]–[26] and show a significantly higher polymerization as well as depolymerization activity in the lamellipodium network compared to the lamellum [7], with a distinct depolymerization peak marking the transition to the lamellum [27]. During cell protrusion, both the lamellipodium and the lamellum translocate from the leading cell edge towards the cell center in a process called retrograde flow, but usually the rate of lamellum movement is several times slower than that of the lamellipodium [7].

### Conceptual framework: array treadmilling

Actin in the leading cell extension is continuously transformed between its two forms, monomeric (G-actin) and filamentous (F-actin), by polymerization and depolymerization [28]. Actin filaments are functionally polar polymers, with their faster growing “barbed ends” (also called “plus-ends”) primarily oriented towards the front and their “pointed ends” (“minus-ends”) oriented towards the back of the cell. Elongating filaments abutting the cell membrane extend the cell boundary, thereby producing forward forces by proposed mechanisms such as the thermal ratchet [29], [30]. Intracellular actin kinetics are tightly controlled by regulatory proteins, a conceptual framework of which is given by the “array treadmilling” model [4] outlined in the following. Figure 2 summarizes the essential features represented in our analytical description.

If all filaments were growing, the monomeric actin pool would rapidly be depleted. Filament growth is thus greatly impeded by rapid binding of capping proteins (e.g., CapZ) to filament plus-ends. The associated loss of growing filament tips is compensated by the nucleation of new growing tips as branches on existing filaments. This process is mediated by the Arp2/3 protein complex and results in a dendritic structure of intertwining filaments which is sufficiently stiff to sustain pressures experienced during cell locomotion [31], [32]. In the front of the network all filament minus-ends are thus bound by the Arp2/3 complex attaching the daughter filaments to their mother filaments (see Small [33] for another opinion). Incorporated in each filament subunit is a molecular timer mechanism. An actin monomer added to a filament plus-end bears an ATP (adenosine tri-phosphate) nucleotide. F-actin hydrolyzes to ADP-Pi-F-actin (ADP: adenosine di-phosphate) and subsequently releases its inorganic phosphate to become ADP-F-actin. The Arp2/3 complex bears adenosine nucleotides which hydrolyze in a similar fashion. The age, or nucleotide state, of actin and Arp2/3 sensitively determines the probability of regulatory molecular events on these building blocks. Debranching, i.e., daughter filament detachment from a mother filament further back in the network, might be associated with ATP hydrolysis on Arp2/3 [34]. The protein ADF/cofilin preferentially binds to ADP-F-actin subunits [35] and destabilizes filaments. Bound ADF/cofilin has been shown to enhance subunit dissociation from the minus-end by a factor of 20–30 [35]. ADF/cofilin in addition is likely to promote filament severing [36]. ADF/cofilin in cells can be deactivated, e.g., by phosphorylation through LIM kinases [37] increasing its unbinding rate from actin filaments. Another important regulatory factor in the back of the network is tropomyosin, which competes with ADF/cofilin for binding to ADP-F-actin [38], stabilizing filaments against ADF/cofilin enhanced severing and depolymerization [23]. At the rear of the actin network extension, highly enriched molecular myosin II motors contract the network [39]. Coupled to this myosin activity is a massive depolymerization of actin [27]. This mode of network disassembly is beginning to be understood molecularly [40], [41] and has been described mathematically [14], [42]. Actin monomers and oligomers released from the network travel to the front presumably by diffusion, but active transport processes have also been hypothesized [43]. Actin monomers can then be reused in promoting cell growth at the leading edge, before which, however, their polymerization competence has to be restored by exchanging their bound ADP nucleotide with ATP nucleotide. This process is catalyzed by tight binding of the protein profilin on G-actin [4]. The re-polymerization of previously released actin monomers closes the array treadmilling cycle.

## Methods

### Modeling scope

As a model system we choose migrating fish keratocytes. Migrating keratocytes are a popular model system due to the steady growth of the exceptionally pronounced cytoskeletal extension in both space and time. The extension is structurally simple compared to other cell types, consisting almost exclusively of the sheet-like network and lacking dynamical processes such as actin ruffling or filopodia formation. It therefore displays array treadmilling in its purest form. Protruding keratocyte cytoskeletal extensions deform only marginally [44], which allows for description as an incompressible network and for the capture of important system features even without the consideration of contractile motor elements. Since almost no movement with respect to the substrate (“retrograde flow”) is observed [44], the modeling of localized substrate adhesions [13]–[15], [42] can likewise be neglected for our purposes. Retrograde flow over local adhesions is a prerequisite for the emergence of differential kinematics [13] and hence a precondition for the formation of a lamellum. All other defining characteristics – structure, molecular composition, and kinetics – emerge already without local adhesions, as we show in this article.

The scope of our calculations is on the central part of the leading cytoskeletal extension of a cell (Figure 1). We ask the following questions:

(A) Which of the lamellipodium-lamellum network properties observed in cells can be reproduced with a minimal model incorporating actin nucleation, growth, capping, and depolymerization, even without friction against local adhesions?

(B) Can we subsume these considered molecular interactions into generalized organizational principles that give rise to the characteristics of lamellipodium and lamellum networks?

(C) Can network decay associated with array treadmilling alone account for the finite size of the lamellum in cells, or are additional mechanisms necessary to break down and depolymerize the network?

### Assumptions

The following sections outline and justify the assumptions of the model. An overview of the most important aspects is given in Figure 2.

#### Geometry

The cytoskeletal protrusion represents the frontmost 10 µm of the cell [45] (Figure 1) and has a height of 170 nm [46]. This height strictly applies to the leading ∼2 µm of the cell, while the height further back may exceed this value [6]. Although effects due to three-dimensionality have been found [47], for technical reasons we stick to the still well-accepted approximation of constant vertical thickness.

#### Diffusion and reaction dynamics

Diffusion of monomeric actin is modeled explicitly. The diffusion coefficient of G-actin is assumed to be independent of network density. In contrast to G-actin, accessory proteins (Arp2/3, plus-end capper, ADF/cofilin, tropomyosin, profilin) are considered to be distributed homogenously. With this we ignore potential depletion effects due to limited pools of accessory proteins. Dynamic processes such as nucleotide hydrolysis, plus-end capping, debranching, and depolymerization are modeled using zero or first order reaction kinetics. This is an accepted simplification although the true dynamics are likely to be more complicated than linear. Plus-end uncapping by membrane-associated factors is not included in our model at this stage. Actin nucleotide hydrolysis and binding of ADF/cofilin and tropomyosin are assumed to proceed in a non-cooperative fashion, i.e. on each subunit individually. F-actin subunits can adopt several states (Figure 1B): ATP-F-actin, ADP-F-actin, ADP-F-actin bound by ADF/cofilin (ADF/cofilin-F-actin), or ADP-F-actin bound by tropomyosin (tropomyosin-F-actin). Upon addition to filament plus-ends, ATP-F-actin subunits are irreversibly hydrolyzed to their ADP-Pi analogs and subsequently release the inorganic phosphate (rate *r_hyd_* for this reaction cascade). ADF/cofilin and tropomyosin can then bind ADP-F-actin in a mutually exclusive manner. While tropomyosin is assumed to stay bound in the time scale of network turnover, we assign an unbinding rate *r_ac_^−^* of ADF/cofilin to account for its deactivation by, e.g., LIM kinases [37]. Arp2/3 complex is assumed to protect filaments from minus-end depolymerization [22]. A prerequisite for depolymerization of a filament is hence debranching, i.e. the detachment of its minus-end from Arp2/3 complex. The exchange on G-actin of ADF/cofilin by profilin and the subsequent nucleotide exchange are not modeled explicitly since these processes have been shown to be sufficiently fast to not significantly affect G-actin re-polymerization [15], [48]. Thus, all G-actin is considered to be effectively bound by profilin and to bear an ATP nucleotide. An appropriate boundary condition for fixing the G-actin profile is the total actin concentration in the cytoskeletal extension, which is experimentally accessible. An alternative but experimentally less well accessible boundary condition would be the G-actin concentration in the convergence zone, *c*(*x* = *L_sys_*).

#### Filament nucleation

Filament initiation mechanisms discussed in literature [49] include nucleation by the Arp2/3 complex, formin, spire, and ADF/cofilin [50], as well as spontaneous nucleation without participation of any nucleator protein [51]. Lacking clear evidence that formins or spire proteins are required for lamellipodial growth, we do not consider these nucleators here. Spontaneous nucleation is likewise ignored. The assumed high cellular concentration of profilin reduces the probability of this process [52], making it irrelevant for the analysis of the system's steady state. We focus on Arp2/3 induced nucleation instead. Following the argumentation by Huber [12] and others [53], [54], we consider Arp2/3 activation the rate-limiting step, which makes the nucleation rate independent from G- and F-actin concentrations.

#### Network properties

We assume actin polymerization to occur exclusively at the leading edge [7]. Elongating filaments have to bend the cell membrane and break attachments between actin cortex and membrane during elongation. We presume that the dependence of the filament elongation rate on the resistance of the cell membrane can be described by a thermal ratchet mechanism [29], [55]. Following Mogilner [30] we assume a filament to be mechanically supported by the surrounding network behind its free leading end, i.e. behind its first attached Arp2/3 complex. With typical nucleation rates (Text S1) and network growth rates, free filament ends on average do not exceed ∼250 nm and are able to support forces on the order of 5 pN [56], which is sufficient to overcome typical membrane resistances of ∼1 pN per filament. We therefore neglect filament buckling. Polymerization has also been measured further back in the cytoskeletal extension, with rates approximately proportional to the steady-state amount of actin filaments [26], which can be interpreted to be due to the creation of filament plus-ends by severing [12]. Filament severing and also annealing are omitted at this stage of the model, which however does not detract from the validity of the results (see our argumentation in the Discussion). Due to the presence of high intracellular concentrations of profilin, filament minus-end growth is neglected [4]. Filaments are assumed to be oriented on average with angles of α = ±35° with respect to the direction of network growth in agreement with electron microscopy data on keratocytes [57] and simulations [58]. In accordance with experimental data on keratocytes [44], we consider the rate of filament transport with respect to the leading edge to be constant throughout the network. Potential reasons why in this sense the keratocyte front appears almost incompressible are not subject of this article. Effects of network compressibility on our results are addressed in the Discussion.

#### Effects of ADF/cofilin and tropomyosin

ADF/cofilin has been shown to bind with high affinity to the ADP-bound forms of actin, but not to the ATP-bound forms, and to greatly enhance actin filament depolymerization [4], [59]. We thus assume ADF/cofilin to bind to ADP-F-actin subunits exclusively and to increase their depolymerization rate from the minus-end 30-fold [12], [35]. We ignore effects of cooperative binding and assume a single binding constant *r_ac_* to ADP-F-actin. Filament severing activity of ADF/cofilin [36] is ignored here but has been addressed by Huber et al. [12]. Tropomoysin, just as ADF/cofilin, is assumed to bind exclusively to the ADP-form of F-actin. It has been shown that tropomyosin stabilizes filaments, effectively reducing depolymerization [60]. Following Huber et al. [12], this effect is modeled by assigning the unaltered depolymerization rate 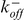 once tropomyosin is bound as opposed to the ADF/cofilin associated, increased depolymerization rate. Tropomyosin and ADF/cofilin binding to F-actin subunits is mutually exclusive in our model. In this competition for actin, we assume that tropomyosin, once bound, stays attached on the time scale of network turnover, in compliance with biochemical experiments [38]. In contrast, ADF/cofilin has a finite unbinding rate in our model, mimicking its deactivation, e.g., by phosphorylation through LIM kinases [37]. This differential treatment of ADF/cofilin and tropomyosin puts the latter at an advantage for binding to actin on the larger time scales of the system, i.e., further back in the network. For technical reasons we assume that tropomyosin binds to ADP-F-actin subunits with a 1∶1 stoichiometry. In fact, a single tropomyosin molecule can bind six or seven adjacent F-actin subunits [61]. We thus explore the lower bound of the actin stabilizing effect.

#### Convergence zone

At the rear of the actin network extension in cells, highly enriched molecular myosin II motors contract the network [39], [62]. Associated with this myosin activity is a massive depolymerization of actin [27]. In order to account for the effect of this “convergence zone” [63], in our model all actin depolymerizes abruptly after 10 µm from the leading edge.

### Model description

The model is implemented as a set of coupled one-dimensional integral and differential equations which describe a treadmilling actin network in steady state. The actin network is characterized in terms of a concentration field, i.e., the mathematical solution does not describe individual filaments. The coordinate axis of the one-dimensional model system (Figure 1) is oriented perpendicular to the leading edge of the cell, with its origin on the leading cell edge and its boundary +∞ behind the cell (“stationary frame”). Reference frames moving in the +*x* direction with the speed of the network will be referred to as “moving frames”.

We define the following spatial properties of the system (in the order of appearance in the text):

*F*(*x*) F-actin concentration (F-actin subunit concentration), µM

*P*(*L,x*) filament length distribution, µm^−1^

*L_mean_*(*x*) mean filament length, µm

*M*(*x*) minus-end concentration (branched and debranched filaments combined), µM

*J_d_*(*x*) depolymerization source density, µM s^−1^

*c*(*x*) G-actin concentration, µM

We define the following state probabilities of F-actin subunits as a function of their *dwell time t_dwell_* in the filament (duration since addition to the filament plus-end) (compare Figure 1B):

*p_atp_*(*t_dwell_*) probability of an F-actin subunit to bear an ATP (adenosine triphosphate) nucleotide

*p_adp_*(*t_dwell_*) probability of an F-actin subunit to bear an ADP (adenosine diphosphate) nucleotide

*p_ac_*(*t_dwell_*) probability of an ADP-F-actin subunit to be bound by ADF/cofilin

*p_tm_*(*t_dwell_*) probability of an ADP-F-actin subunit to be bound by tropomyosin

We define the following state probabilities of filaments as functions of the time *t* since nucleation:

*p_deb_*(*t*) probability that a filament has debranched

*p_uc_*(*t*) probability that a filament's plus-end is still uncapped

All model assumptions are justified in the previous section. Model parameters based on biological literature are summarized in Table 1. Explanations of the choices of model parameter values are given in Text S1. Figures 1, 2, and S1 provide visual support for understanding the following derivations.

**Table 1:** Definition of model parameters based on biological literature.

Parameter	Value	Description	Source
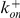	12 µM^−1^ s^−1^	On-rate constant of ATP-actin monomers to uncapped plus-end	[4]
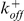	1.4 s^−1^	Off-rate of actin subunits from uncapped plus-end	[4]
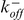	0.3 s^−1^	Off-rate of ADP-actin subunits from minus-end	[4]
*s_ac_*	30	Off-rate enhancement for ADP-actin subunits from minus-end when bound by ADF/cofilin	[35]
*L_sys_*	10 µm	Length of lamellipodium/lamellum	[45]
*h*	0.17 µm	Mean height of lamellipodium/lamellum	[46]
*δ_p_*	2.2 nm	Filament length increment in *x* direction per subunit	[58], [85]
*D*	5 µm^2^ s^−1^	G-actin diffusion coefficient	[67]
*A*	350 µM	Total actin concentration (average over 0<*x*<*L_sys_*)	[12]
*B*	440 µM	Concentration of growing plus-ends at the leading edge	[86]
*F_mem_*	100 pN µm^−1^	Membrane resistance force per unit edge length	[48]
*r_cap_*	1 s^−1^	Plus-end capping rate	[87]
*r_ac_*	0.5 s^−1^	ADF/cofilin binding rate to ADP-F-actin	[12]
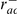	0.2 s^−1^	ADF/cofilin unbinding rate	[12]
*r_tm_*	0.2 s^−1^	Tropomyosin binding rate to ADP-F-actin	[12]
*r_hyd_*	0.3 s^−1^	ATP hydrolysis rate on F-actin subunits	Derived from Svitkina et al. [22]
*r_deb_*	0.5 s^−1^	Debranching rate	Derived from Svitkina et al. [45]

See Text S1 for detailed explanations.

#### Nucleation, growth, and depolymerization of filaments

Actin filaments are nucleated with constant rate *N_0_* at the system boundary representing the cell's leading edge (*x* = 0). Actin monomers add to filament plus-ends in a concentration dependent manner and also dissociate, leading to a net rate of monomer addition to a filament's plus-end (*plus-end rate*)
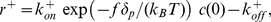
(1)where 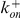
and 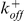 the on- and off-rate constants at the plus-end, 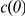 is the monomeric actin concentration at *x* = 0, *k_B_* is the Boltzmann constant, *T* is the temperature, *f* is the force acting on a single filament in the *x* direction, and ∞*δ_p_* is the projected filament length increment in the *x* direction per added monomer. The exponential accounts for the dependence of the on-rate on the resistance experienced by elongating filaments in the framework of a thermal ratchet model. The boundary monomer concentration *c*(*0*) in Equation (1) is the decisive unknown variable that has to be determined by solving the coupled system of all following equations. The plus-end rate 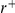 defines the *network growth rate* (filament transport velocity)
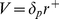
(2)with which the polymerizing network pushes itself off the cell membrane. Taking the state conversions of F-actin into account (Figure 1B), the *state probabilities of an F-actin subunit, p_atp_*(*t_dwell_*), *p_adp_*(*t_dwell_*), *p_ac_*(*t_dwell_*), and *p_tm_*(*t_dwell_*), as functions of its dwell time *t_dwell_* in the filament are solutions of the coupled system of differential equations
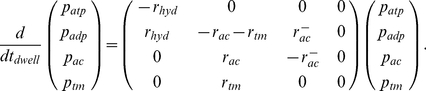
(3)


Subunits dissociate from free minus-ends with a rate which is a function of the filament minus-end state: 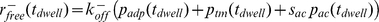. The factor *s_ac_*>1 accounts for the increased subunit dissociation upon ADF/cofilin binding. Bound tropomyosin brings the dissociation rate back to its in vitro value 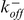, mimicking its actin stabilizing effect. 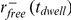 refers to minus-ends of debranched filaments, i.e. of filaments which have detached from the mother filament. Filament debranching, the prerequisite for filament depolymerization, is modeled as a Poisson process with rate *r_deb_*, yielding a probability that a daughter filament has separated from its mother filament of 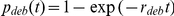, where *t* is the time since nucleation of the filament. The *minus-end rate* 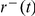 is derived by multiplication of the free minus-end rate 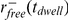 with this debranching probability. As argued in Text S2, the dwell time *t_dwell_* can hereby be approximated by *t*, yielding a minus-end rate 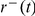 which only depends on *t*: 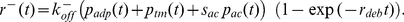
(4)


Equations (1) and (4) describe the rates of filament length change at the filament plus- and minus-end and can be used to derive the length of a filament as a function of time.

#### Plus-end filament capping

Elongation of a filament ceases after irreversible capping of its plus-end. Each filament's history is unambiguously described by the time the filament stayed uncapped, *t_uc_*, and the subsequent time duration that it has been capped, *τ_c_*. The network consists of filaments with all combinations of *τ_c_* and *t_uc_* (Figures 1, S1). Capped filaments are transported backwards within the network with rate *V*. Filaments with the same *τ_c_* therefore share the same plus-end position and will henceforth be identified as a “group”. There is a continuous distribution of capped times *τ_c_* and thus an infinite number of groups. The *length of a filament, L,* is related to its capped time *τ_c_* and uncapped time *t_uc_* by
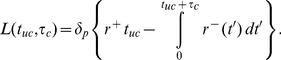
(5)


While the first term represents plus-end filament growth, which is limited to the uncapped phase, the second term attends to minus-end shrinkage. Stochastic plus-end capping is modeled as an irreversible first order reaction with capping rate *r_cap_*, yielding *p_uc_*(t)  =  exp(-*r_cap_ t*) as the probability that a filament is still uncapped. The *fraction of filaments of a group getting capped between t_uc_ and t_uc_+dt_uc_* is derived from this as
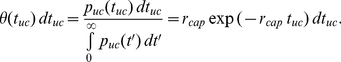
(6)


The *plus-end density at the leading edge B* (in µM) is the sum of all density contributions of plus-ends nucleated during time spans of length *dt_uc_*, *N_0_ dt_uc_*, weighted by their probabilities *p_uc_*(*t_uc_*) of still being uncapped after time *t_uc_*:
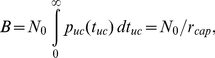
(7)where *N_0_* is the steady state nucleation rate at the leading edge (in µM s^−1^). The force *f* on a single filament used in Equation (1) is constant and is related to *B* by *f* =  *F_mem_*/(*Bh*δ*_p_* *η)*, where *F_mem_* is the membrane resistance force per unit edge length, *h* is the mean height of the cell front, and *η* =  602.2 µM^−1^ µm^−3^ is the conversion factor between the density units µM and µm^−3^.

#### Network density

The concentration profile of filamentous actin, *F*(*x*), is derived by examining how many filaments of each group cross position *x*. Figure S1 illustrates the following derivation with two exemplary filament groups 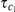
(left box) and 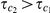 (right box). Since all filament plus-ends of a group *τ_c_* are at position *V τ_c_*, we have to determine how many of the filaments of this group have been uncapped long enough - before their inevitable capping - to exceed the minimal length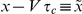. The *actin concentration of a group τ_c_* (in µM s^−1^) at a distance 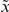 from the group's plus-ends is therefore given by
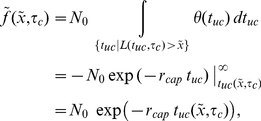
(8)where *N_0_* is the concentration of filamentous actin emerging at the leading edge per time. Figure S1 illustrates how these values correspond to areas under *t_uc_*-θ curves. The integration range includes all uncapped times *t_uc_* for which the filament length *L*(*t_uc_, τ_c_*) exceeds the minimal probe length 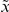
(note that *τ_c_* is constant here and represents the current filament group). For physiological minus-end depolymerization rates, 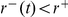 always holds and hence *L*(*t_uc_, τ_c_*) increases monotonically with *t_uc_* (see the *t_uc_*-*L* curves in Figure S1). The integration range is thus from 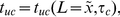
(9)determined by Equation (5), to infinity. 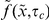
represents the group contribution to the filament density in the moving frame. In the stationary frame, this contribution is
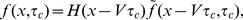
(10)where H(*x*) is the Heaviside function. The *concentration of F-actin with capped plus-ends*, *F_c_*(*x*), is retrieved by integrating all capped (0<*τ_c_*<∞) group contributions *f*(*x, τ_c_*),
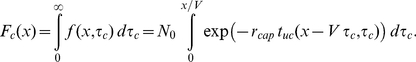
(11)


Besides the unlimited number of groups of capped filaments (0<*τ_c_*<∞) there exists one group of uncapped filaments. The F-actin concentration at the plus-end position of this group is the plus-end density at the leading edge (in µM), *B* = *N_0_*/*r_cap_* (Equation 7). The *concentration of uncapped F-actin*, *F_uc_*(*x*), is then given by
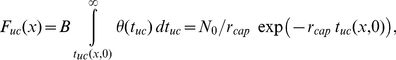
(12)which together with the contribution of capped filaments yields the *total F-actin concentration F*(*x*):
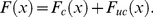
(13)


#### Filament length distribution

The following derivation holds for both capped (*τ_c_*>0) and uncapped (*τ_c_* = 0) filaments. The fraction of filaments of a group *τ_c_* which have a length *L* is given by 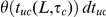, with the argument *t_uc_* calculated from Equation (5). Filaments with length *L* make up the fraction
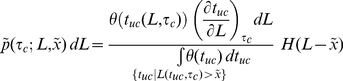
(14)of all of this group's filaments exceeding a length 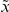. Transformation into the stationary frame analogous to Equation (10) yields 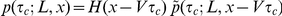 as the probability (in µm^−1^) that a filament of the group *τ_c_* which transects position *x* has length *L*. The *filament length distribution at position x* under consideration of all groups, *P*(*L,x*) (in µm^−1^), is the weighted sum of the contributions of the individual groups, *p*(*τ_c_; L, x*), where the weight coefficients equal each group's share of the total filamentous actin concentration at this position, (*f*(*x, τ_c_*) *dτ_c_*)/*F*(*x*):
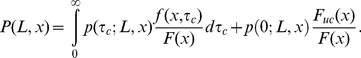
(15)


Here, the last term accounts for the single group of uncapped filaments, which has to be considered separately.

The *mean filament length at position x* is the sum of all possible filament lengths, weighted by the probabilities of their occurrence: 
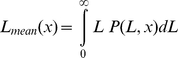
(16)


#### Network decay

Filaments debranch and depolymerize, transferring subunits from the filamentous to the monomeric actin pool. Actin dissociates from minus-ends of filaments. The *minus-end concentration profile of a group* (in µM s^−1^) is given by 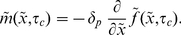
(17)


Transformation into the stationary frame analogous to Equation (10) and integration over all groups yields the *total minus-end concentration profile*

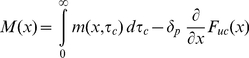
(18)with 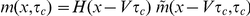. Here, the first and the second term represent the minus-end concentration contributions of capped and uncapped filaments, respectively.

The *actin depolymerization source density J_d_*(*x*), i.e., the number of actin monomers per time and volume entering the monomeric actin pool at position *x* (in µM s^−1^), is the product of the minus-end concentration *M*(*x*) and the minus-end depolymerization rate 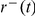 at this position: 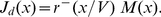
(19)


#### Monomer diffusion

The *concentration profile of monomeric actin*, *c*(*x*), is described by the diffusion equation with depolymerization source density *J_d_*(*x*) as additive term accounting for the coupling between the F- and the G-actin pool: 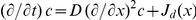. In the stationary case, 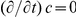 and thus 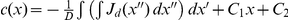. F-actin subunits reaching the system boundary (*x*  =  *L_sys_*) in the convergence zone depolymerize, causing an influx of actin into the monomer pool at this position and a corresponding G-actin concentration gradient
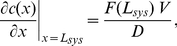
(20)which fixes *C_1_*_._ We assume that the system contains a fixed amount of total (F- and G-) actin and use
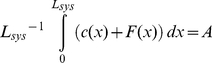
(21)to fix *C*_2,_ where *A* is the average total actin concentration in the system. The free variable *c*(0), the monomeric actin concentration at *x* = 0, can then be determined by solving the implicit Equation (21).

We distinguish the two scenarios of a confined and an unconfined system. We define a confined system as one where the actin network length is restricted by the system size and F-actin hence effectively depolymerizes in the convergence zone (*F*(*L_sys_*)>0 in Equation (20)). This situation applies to crawling cytoplastic fragments [39] and to cell lamella/lamellipodia [27]. We also consider unconfined systems (Results section “Unconfined treadmilling”), where we choose the system size *L_sys_* sufficiently large to allow a drop of F-actin concentration well before the system boundary (*F*(*L_sys_*) = 0), which for example can be used to describe spherical network growth from beads in a bulk phase [64].

### Model solution

The equations of the model were discretized and solved numerically, using parameter values in agreement with literature (Table 1). Starting with the G-actin concentration at the leading edge, *c*(0) in Equation (1), the coupled equations eventually return an output value for *c*(0) with Equation (21). A consistent set of solution functions was retrieved by iteratively adjusting the *c*(0) input value until the resulting *c*(0) output value differed from the *c*(0) input value by less than 50 nM. All integrations were carried out using the trapezoidal rule. All differentiations were carried out using backward differencing. We found a discretization of 0.02 µm and 0.01 s in space and time coordinates, respectively, to be sufficient to numerically reproduce analytical predictions (derived in Text S3) with adequate precision (less than 5% deviation in all data; Figure S2). For the treatment of unconfined systems, spatial discretization was chosen coarser for efficient computing but never exceeded 0.1 µm.

## Results

### Reproduction of lamellipodium and lamellum network properties

**Figure 3:**
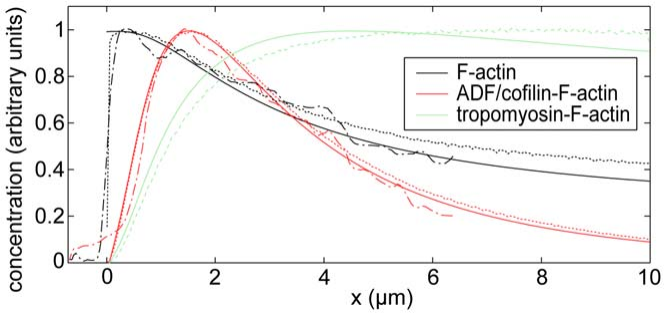
Concentration curves for F-actin, ADF/cofilin-F-actin, and tropomyosin-F-actin. The calculated curves (solid lines) are normalized to compare results with cell experimental (dash-dotted lines) and simulation (dotted lines) fluorescence curves. ADF/cofilin decorated F-actin dominates within the first ∼2 µm, whereas for x≥2 µm, tropomyosin is the dominating element. Calculated F-actin as well as ADF/cofilin-F-actin signals are in good agreement with data from Svitkina and Borisy [22] depicted as dash-dotted lines. The calculated signals also agree reasonably well with simulation data from Huber et al. [12] drawn as dotted lines (<25% deviation at each data point).

We obtain G-actin concentrations at the front of *c(0)* ∼15 µM (Figure 1C). These concentrations give rise to network growth rates *V* between 10 and 15 µm/min (Figure S3), in agreement with values observed for moving keratocytes [31]. We obtain leading edge F-actin concentrations of ∼0.5 mM (Figure 1C) and a decay of this quantity towards the back of the cell matching well known characteristics for keratocytes and other cell types [22], [24], [45] (Figure 3).

#### Structure

**Figure 4:**
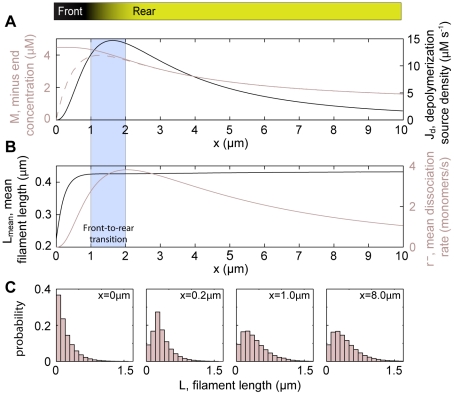
Model solutions describing essential lamellipodium (“front”) and lamellum (“rear”) characteristics. These characteristics are a local maximum of the depolymerization source density *J_d_* at the transition between front and rear (A) and a distinctive filament length distribution *L_mean_* with its steep gradient within the front and a plateau in the rear (B). (C) Filament length histograms at different distances from the leading edge illustrate the emergence of the mean filament length distribution in (B). The dashed light line in (A) represents the debranched fraction of all (solid light line) minus-ends. *r* ^−^ in (B) is the ensemble-averaged rate of subunit loss from a filament minus-end at position *x*.

The mean filament length is short at the extreme leading edge of our model system and rapidly increases to eventually reach a constant value after ∼1 µm (Figure 4B). A steep gradient in filament length has been observed for various cell types [4], [22], [65]. Electron microscopy has revealed filament lengths on the order of few hundreds of nanometers at the cell membrane as well as a restructuring of the network and a steep increase of filament lengths already within few microns from the leading edge [45], both in agreement with our results. The different filament length characteristics within cells are one of the criteria commonly used to distinguish between the lamellipodium, with its short filaments at the very front, and the lamellum, which consists of long filaments exclusively [6], [24]. The sudden increase of the mean filament length in the frontal zone can be understood by analyzing the filament length histograms at various distances from the leading edge (Figure 4C). Since capping is modeled as a Poisson process, the filament length distribution at *x* = 0 falls off exponentially, and most filaments at the leading edge are short. Many of these short filaments depolymerize completely soon after capping, rendering the length distribution non-exponential and effectively increasing the mean filament length already close behind the leading edge.

#### Kinetics

Figure 4A shows calculated distributions of the depolymerization source density *J_d_*(*x*), i.e. the actin concentration transfer per time from the filamentous to the monomeric actin pool. A maximum of *J_d_*(*x*) at *x* ∼1-2 µm is in agreement with speckle fluorescence data on migrating epithelial cells [7], [27]. Ponti et al. [7] have identified a net network disassembly peak marking the beginning of the transition from lamellipodium to lamellum, which is reproduced by our model. We can understand the occurrence of our depolymerization peak by analyzing the mean dissociation rate profile (Figure 4B), i.e., the ensemble-averaged rate of subunit loss from a filament minus-end, *r* ^−^ (*x*/*V*). *J_d_*(*x*) is the product of this quantity and the concentration of depolymerization sites at this position, i.e., of filament minus-ends, *M*(*x*) (Figure 4A, Equation 19). The initial rise of *J_d_* is mainly due to the increase in *r* ^−^ reflecting debranching and ADF/cofilin binding. With increasing *x* and thus continuing time, the mean dissociation rate decreases again owing to replacement of actin bound ADF/cofilin by stabilizing tropomyosin. As a result, the depolymerization source density *J_d_*(*x*) declines for *x*≥2 µm. This decline is however not determined by *r* ^−^ exclusively but also by *M*; in the course of time more and more free minus-ends vanish due to complete depolymerization of filaments. Depolymerization hence in part deprives itself from its basis by eliminating free minus-ends. This self-induced decline in depolymerization is seen most clearly in a simplified hypothetical scenario of immediate hydrolysis, debranching, and ADF/cofilin binding in the absence of tropomyosin. Here, the mean dissociation rate is constant and therefore does not affect the profile of *J_d_*, the drop of which is solely the result of the loss of free minus-ends towards the back (Figure S2B).

#### Molecular composition

As a consequence of the implemented competition between ADF/cofilin and tropomyosin for binding to F-actin, we observe a crossover in the signals of F-actin and ADF/cofilin-F-actin, where ADF/cofilin is increasingly removed from the network (Figure 3). Concomitantly, while ADF/cofilin-F-actin dominates the frontal zone up to ∼2 µm from the edge, tropomyosin-F-actin prevails further back. Elevated levels of network-bound ADF/cofilin and tropomyosin have been found for the lamellipodium [22], [25] and the lamellum [23], [25] in cells, respectively. Tropomyosin is known to inhibit Arp2/3 induced branching [66]. Its prevalence in the back of the network hence could account for the rather unbranched architecture of the lamellum [45]. For these reasons, tropomyosin can be considered a marker for the lamellum. Our calculated distributions of tropomyosin and ADF/cofilin therefore reproduce the characteristic molecular composition of the lamellipodium and the lamellum in various cell types. The fact that we obtain a separation of ADF/cofilin and tropomyosin might not seem surprising since a separation mechanism is a direct input of our model. It is interesting, though, that choosing experimentally verified reaction rates we get quantitative agreement with experimental data as to where the transition in molecular composition is located.

The criteria of filament length gradient, depolymerization peak position, and ADF/cofilin-tropomyosin separation are commonly used by experimentalists to distinguish between the lamellipodium and the lamellum. With our model we thus reproduce essential characteristics of the leading cell front. Differential kinematics are not among our reproduced cell characteristics, due to the absence of local adhesions in our model (see the Discussion). Filament severing is likewise not included in the model. Our previous simulations, however, confirmed the conservation of the observed characteristics in the presence of severing [12].

One could assume that tropomyosin, due to its filament stabilizing function, were required for the transition of the lamellipodium to the long-filament network of the lamellum. In contrast, even in the absence of tropomyosin we obtain a steep gradient in filament lengths (Figure S2B) and a peak in depolymerization source density (Figure S3). Tropomyosin is obviously not necessary for the formation of these essential features of the lamellipodium-lamellum system. Filament stabilizers such as tropomyosin have important regulatory roles, however, discussed in Text S4.

The results obtained here agree well with those of our Monte Carlo simulations [12]. While this is reassuring, the analytical formulation more importantly also refines and generalizes our understanding of the mechanisms underlying network feature emergence, as will now be developed.

### Generalized organizational principles

**Figure 5:**
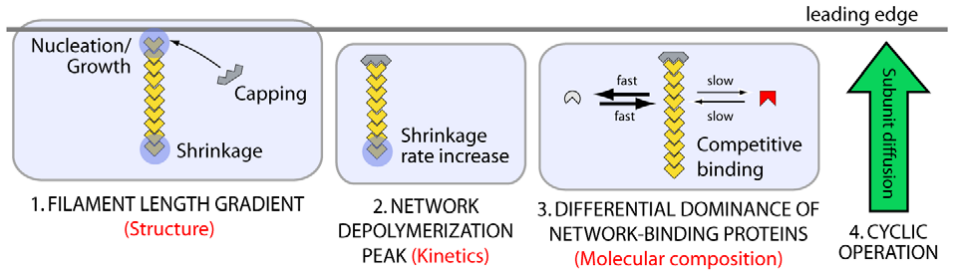
General organizational principles giving rise to lamellipodium and lamellum characteristics of the cell front. 1: Nucleation, growth and stochastic capping on one end and shrinkage on the other end of a filament suffice to create a characteristic filament length gradient at the tip of the lamellipodium (Figure 4B). 2: A gradual destabilization of filaments with time creates a local maximum in network depolymerization (Figure 4A). 3: A differential dominance of network-binding proteins (Figure 3) can be achieved via competitive binding of a fast and a slow binding/unbinding protein. 4: Subunit diffusion to the front closes the subunit cycle, allowing cyclic operation.

In the previous section we have identified molecular mechanisms responsible for the emergence of essential characteristics of the lamellipodium-lamellum system. We now subsume these mechanisms into generalized organizational principles (Figure 5). We hence elevate the model from a particular molecular implementation and distil the minimal requirements giving rise to a migrating cell front with structural, molecular, and kinetic properties of a lamellipodium and lamellum.

At the root of cell front organization is the internal polarity of the treadmilling machinery, with nucleation taking place exclusively at one side and the polymerization constantly driving the network along one specific direction. Temporal fingerprints of processes on filament subunits are thus inevitably translated into spatially separated system characteristics.

A first principle now gives rise to the filament length gradient in the very front (lamellipodium) of the cell. The only ingredients needed to create this characteristic are filament nucleation and growth, stopped by stochastic capping, on the front end and some continuous filament shrinkage on the other end. Filament lengths then do not monotonically decrease with time, i.e., distance from the nucleation zone as one could be tempted to think. Instead, the complete depolymerization of the many rapidly capped and thus short filaments localized close to the nucleation site produces a steep increase of the mean filament length.

A second organizational principle creates the local network depolymerization peak (local maximum of the depolymerization source density *J*(*x*)) marking the transition of the lamellipodium into the lamellum. Surprisingly, a gradual destabilization of filaments with time (and thus with distance) suffices. One is tempted to assume that for an increase of the depolymerization source density to be local, filaments would need to be stabilized farther back in the system again. This is not the case, since network depolymerization deprives itself from its basis by eliminating filaments, and therefore eventually will always drop to zero.

A third organizational principle gives rise to the differential dominance of network-binding proteins in cells, as exemplified as a spatial separation of ADF/cofilin and tropomyosin. This can be achieved with a simple mechanism of competitive binding. If a factor with quick binding/unbinding rate and a factor with slow binding/unbinding rate compete for a filament subunit, the fast factor will dominate on short time scales (i.e., close to the edge), but since this factor also unbinds quickly, the slow factor will eventually prevail, and hence dominate on larger time scales on (i.e., in the back).

Fourth, a feedback mechanism is necessary which closes the subunit cycle by bringing subunits which have left filaments back to the nucleation zone, for another round of filament elongation. Because the first organizational principle creates a subunit gradient, this step is automatically achieved by means of diffusion. Other mechanisms, such as active transport by motors or by gel contraction, can just as well fulfill this task.

In conclusion, a strikingly simple set of non-equilibrium interactions can already produce a steady state describing the treadmilling machinery with essential lamellipodium and lamellum characteristics. This steady state is furthermore produced reliably, regardless of the specific choice of parameters, as parameter scans show (Figure S3, Text S4).

### Unconfined treadmilling

So far we have assumed rapid depolymerization of F-actin when it reaches the rear boundary at *x* = 10 µm, thus rigorously limiting network size to cell lamellum dimensions. We now ask how network treadmilling behaves without this myosin-associated depolymerization mechanism in the convergence zone of cells. The question is whether we can even then obtain network lengths sufficiently small to account for the leading cytoskeletal extension in migrating cells, and if so, whether cells operate in the respective regimes. This question could be addressed only with our analytical approach, not with the previously published computer simulations [12], due to a calculation time reduction by one to two orders of magnitude with the new method.

**Figure 6:**
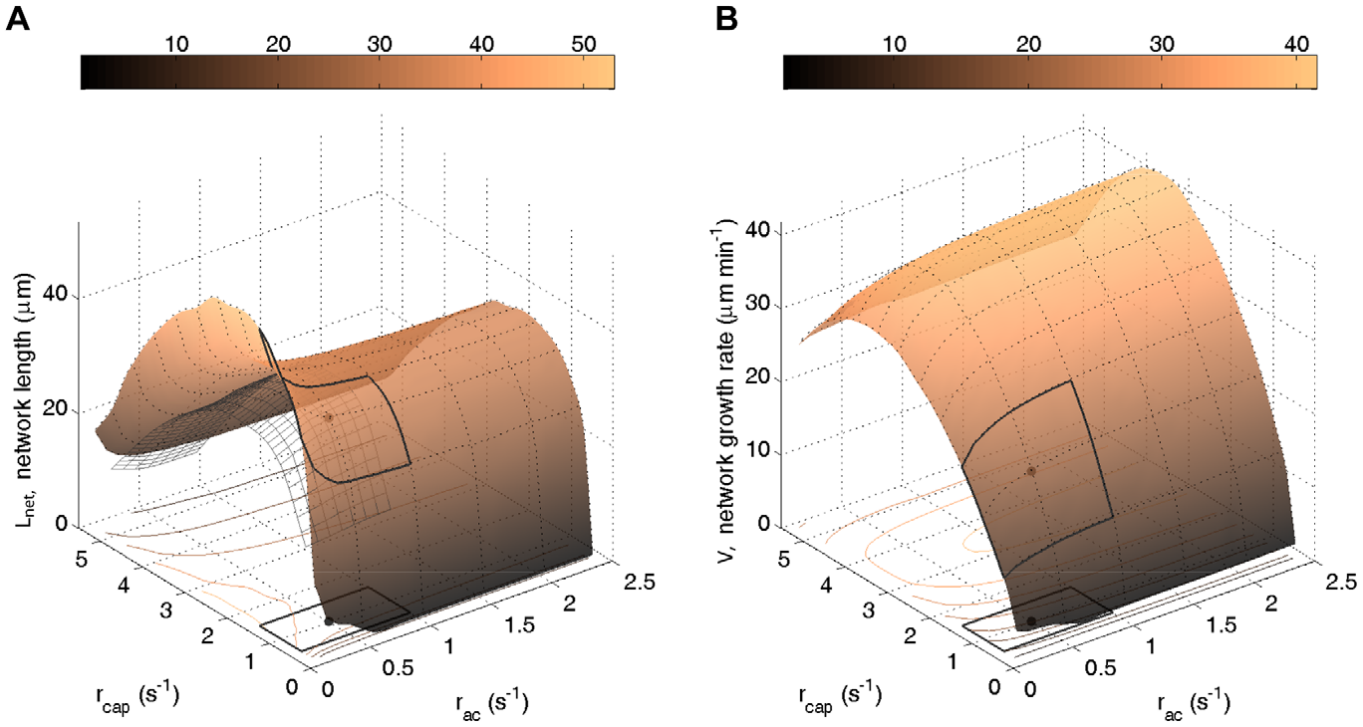
Network length and network growth rate of a rearwards unconfined network. Network length *L_net_* (A) and network growth rate *V* (B) are plotted against plus-end capping rate *r_cap_* and ADF/cofilin binding rate *r_ac_* in the absence of tropomyosin (*r_tm_* = 0 s^−1^). Parameters plausibly applying to living cells (0.03 s^−1^<*r_ac_*<1 s^−1^, 0.5 s^−1^<*r_cap_*<1.5 s^−1^) are encircled with black rectangles. The mesh plot in (A) represents the case of neglected ADF/cofilin deactivation (*r_ac_* ^−^ = 0 s^−1^), while *r_ac_* ^−^ = 0.2 s^−1^ otherwise. 15×13 data points were calculated and spline-interpolated for each plot.

For exploring the lower bound of attainable network lengths, tropomyosin binding, which extends the network, is disabled. Following Novak et al. [47], we assume nucleotide exchange on G-actin monomers to be fast and consequently a variation in its rate to have only little effect on the supply of polymerization-competent G-actin at the front; we therefore omit profilin concentration variations in this study. Calculated network lengths decrease only marginally when filament debranching and actin nucleotide hydrolysis are assumed to happen instantaneously rather than with the rates in Table 1 (data not shown). Candidates with significant impact on network length turn out to be ADF/cofilin binding and unbinding as well as plus-end capping. Figure 6 shows the network length and the network growth rate as functions of the ADF/cofilin binding rate *r_ac_* and the capping rate *r_cap_*. The network length is defined as the distance from the leading edge where the F-actin concentration drops below 5 µM (Text S1). While the capping rate affects treadmilling significantly, the impact of ADF/cofilin binding rate modulations is rather moderate. These trends can be understood as follows.

With increasing capping rate, the decrease of the number of pushing filaments reduces G-actin monomer consumption and thus allows higher G-actin concentrations at the front. As a result, network growth rate and network length increase. However, more rapidly capped filaments are also much shorter on average and therefore vanish earlier by depolymerization, causing a decline of the network length starting with *r_cap_* ∼2 s^−1^. With fewer filaments pushing, each filament also experiences a higher force, eventually leading to a network slowdown by the Brownian ratchet mechanism for capping rates *r_cap_*>3.5 s^−1^ (Figure 6B), which enhances the network shortening effect at these high capping rates.

An increase of the ADF/cofilin binding rate causes a moderate monotonic decrease of network length due to enhanced depolymerization of filaments. At the same time the network growth rate increases owing to enhanced supply of G-actin monomers freed from the network. Without this speed-up of network advancement, the shortening effect of ADF/cofilin would affect the network length more drastically.

A noticeable network shrinkage can be achieved by switching off ADF/cofilin deactivation (*r_ac_* ^−^ = 0 s^−1^; Figure 6A, mesh). This effect is restricted to small ADF/cofilin binding rates (*r_ac_*≥0.5 s^−1^). For *r_ac_*>0.5 s^−1^, unbinding events barely affect network kinetics due to rapid rebinding of an ADF/cofilin to any previously freed F-actin subunit.

The analysis shows that with parameters which plausibly apply to cells (0.03 s^−1^<*r_ac_*<1 s^−1^, 0.5 s^−1^<*r_cap_*<1.5 s^−1^, discussed in Text S1; Figure 6 black rectangles), calculated network lengths clearly exceed cell lamellum dimensions (10 µm), regardless of if ADF/cofilin deactivation is taken into account or not. In the presence of tropomyosin, treadmilling networks are expected to be even longer (data not shown). In order to reduce network dimensions, the cell could operate at very low capping rates (*r_cap_*<0.3 s^−1^). This, however, would be accompanied with a depletion of the monomer pool by the multitude of pushing filaments and hence with network growth rates which are too small for the cell to fulfill its physiological functions (Figure 6B). Very high capping rates (*r_cap_*>5 s^−1^) are another scenario which creates short networks in the framework of our model. As the capping rate increases, however, network concentration and filament lengths decrease, and our assumption of network incompressibility will become invalid. With typical network growth rates of ∼15 µm/min and network concentrations of ∼250 µM (corresponding to mesh sizes of ∼50 nm), filament lengths will drop below the mesh size for capping rates *r_cap_*>5 s^−1^, which thus represents an upper limit for the mechanical integrity of the network and all the more so for its incompressibility.

**Figure 7:**
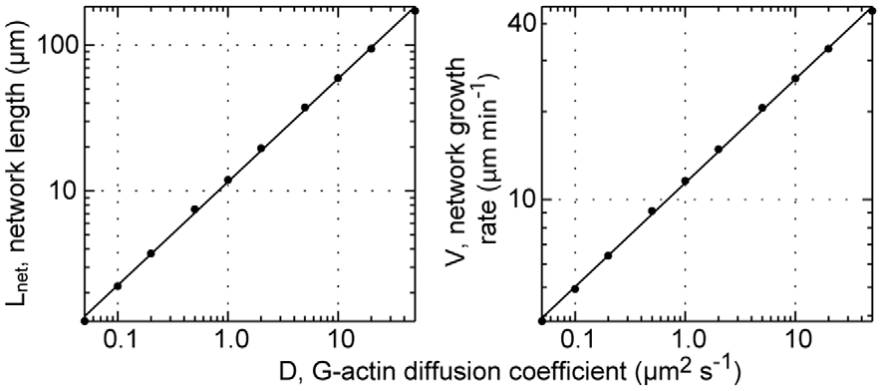
Length and growth rate of a rearwards unconfined network. Network length *L_net_* (left) and network growth rate *V* (right) are plotted against the diffusion coefficient *D* of actin monomers (dots: numerically calculated data; lines: exponential fits) in the absence of tropomyosin (*r_tm_* = 0 s^−1^). *L_net_* and *V* follow power laws with exponents 0.71 and 0.36, respectively.

Since each subunit freed from the network must diffuse to the front before re-incorporation into the network, the diffusion coefficient for G-actin monomers in the lamellum cytoskeleton will sensitively affect network treadmilling. We ask if diffusion can limit network dimensions to values compatible with those observed in cells. The network length and the network growth rate as functions of the diffusion coefficient follow scaling laws with exponents 0.715 and 0.356, respectively (Figure 7). To produce lamellum-sized networks, the G-actin diffusion coefficient would have to be as small as 0.8 µm^2^ s^−1^. Such low values, however, are almost an order of magnitude below estimates for G-actin in the cytoplasm (5–6 µm^2^ s^−1^, [67]) or lamellipodia/lamella [43] based on photobleaching experiments. A value of *D* = 5 µm^2^ s^−1^ produces network lengths of ∼40 µm even without the elongating effect of tropomyosin. Zicha et al. [43] have measured yet more rapid superdiffusive G-actin transport, which can potentially be explained with hydrodynamic flow induced by actomyosin-contraction in the convergence zone. From our results we conclude that G-actin transport is unlikely to limit network size in cells to lamellum dimensions.

While the assembly of actin at the cell front has been subject to extended experimental and theoretical investigation, how network growth is limited in the back of a cell is still poorly understood. We show here that neither depolymerization enhancement by ADF/cofilin, nor enhanced or reduced filament capping, nor limited diffusion suffice to shorten networks to dimensions of the cell while at the same time maintaining physiological growth speeds and network mechanical integrity. As possible solutions of the problem, filament severing [50] and burst disassembly from filament ends in the presence of Aip1 and Coronin [68] have been identitified. Another attractive lamellum disassembly mechanism is myosin contraction-associated depolymerization in the convergence zone marking the transition between lamellum and cell body [40], which was chosen in this study to limit network growth. Network disassembly due to myosin-induced contraction has been described mathematically [14], [42]. In a zero-order approach, we restrict our phenomenological modeling of the convergence zone to instantaneous network depolymerization at the system's rear boundary.

## Discussion

We have developed an analytical description of the treadmilling actin array which constitutes the leading cytoskeletal extension of motile cells. Our model includes network nucleation, polymerization, and decay, modulated by the binding and unbinding of the network stabilizing and destabilizing factors ADF/cofilin and tropomyosin, as well as diffusion of monomeric actin to the leading edge for re-polymerization. We implemented the model as a set of integro-differential equations, which we solved numerically and in part analytically. We could reproduce actin concentration profiles and growth rates in accordance with cell experiments [7], [22], [27], [45] and computer simulations [12], using parameter values from literature. Moreover, we could show the emergence of distinct structural, kinetic, and molecular characteristics of the treadmilling machinery.

A key result is that a surprisingly small set of functional components that are molecularly realizable in a multitude of ways can, by means of self-organization, reliably create the treadmilling cell machinery with the aforementioned characteristics. First, growth and capping of filaments at one end paired with depolymerization from the other end suffices to create the characteristic filament length gradient at the very front of the system. Second, a gradual filament destabilization with time, implemented for instance by filament debranching, inevitably translates into a localized maximum of network depolymerization a few microns behind the leading edge. Finally, a differential dominance of network-binding proteins can be achieved via competitive binding of a fast and a slow binding/unbinding protein. These three characteristics are accepted necessary criteria distinguishing the lamellipodium and the lamellum of migrating cells.

The act of formulating the model as analytical expressions helped us in decoding the minimal requirements for the emergence of these criteria more precisely than with our former computer simulations [12] and lead to a generalized understanding of cell front organization untied from the specifics of any cell type's molecular inventory. The same function (filament nucleation, stabilization, etc.) could be realized in different cell types by different molecular players. Due to their emergence from very general mechanisms, the observed characteristics can plausibly be assumed by a cell with little molecular or evolutionary costs, and we hence speculate that these organizational principles be conserved across species quite generally. Interestingly, in the light of our results – and contrary to recent suggestions [69]–[71] – proteins of the formin family are in principle not necessary for the formation of lamellum characteristics. Instead, one single nucleation mechanism suffices, e.g., mediation by the Arp2/3 complex.

Naturally, our model bears limitations. In many cells, lamellum retrograde flow is significantly slower than lamellipodium retrograde flow [7]. We did not try to model these differential network kinematics but for technical reasons assumed incompressibility of the network. A possible future extension of the model is the inclusion of network compressibility and local adhesions, which clearly influence dynamics of, e.g., motile neuronal growth cones, fibroblasts, and unpolarized keratocytes. While the lamellipodium in cells is only weakly adherent, strong adhesions commonly begin at the junction to the lamellum [7]. Plausibly, the flow rate of a compressible network will locally decrease in front of focal adhesions, creating the differential network kinematics. A network thus slowed down should also locally compress and result in an overall sharper local depolymerization maximum. Additionally, local stretching stresses between adhesions could promote filament severing by proteins such as ADF/cofilin or gelsolin, as modeled by Shemesh et al. [13]. The fate of filaments after such severing is speculative. Rapid depolymerization by the cooperative activity of cofilin, coronin, and Aip [72] could add to the local depolymerization maximum. Alternatively, rapid short-time filament elongation at exposed plus-ends after severing [12] could support actin re-assembly into, e.g., arclike actin bundles often observed in the lamellum [73]. These views suggest a fortification of our derived characteristics if local substrate adhesions were incorporated in our model. Our conclusion is that while local substrate adhesions are compulsory for differential network kinematics, they are not required for the emergence of the structural, kinetic, and molecular characteristics of the lamellipodium-lamellum system.

Another key finding of this work is robustness, i.e., the development of the treadmilling machinery with its distinct signatures irrespective of the choice of parameters (Figure S3, Text S4). Once a set of proteins implementing the few required functional components is brought together, these proteins will inevitably organize into the motile apparatus.

Despite the fact that the choice of parameters does not affect the existence of the network characteristics, their specifics can plausibly vary with the molecular basis provided by a cell. We show that filament stabilizers such as tropomyosin are not necessary for the creation of the obtained lamellum characteristics but, together with filament stabilizers (e.g., ADF/cofilin), regulate important topological and dynamic properties of the system. An increase in tropomyosin activity, just as a decrease in ADF/cofilin activity, results in a shortening of the lamellipodial zone, a slowdown of array treadmilling (kinematics and network turnover), and an increased fraction of polymerized actin. This is in agreement with cell perturbation experiments by various authors [24], [25], [74]–[77].

The identified organizational principles inevitably terminate the network at its rear end, due to aging-induced depolymerization of the network. To test if these network decay mechanisms are sufficient to also “trim” the machinery to cell sized dimensions, we have modified kinetic model parameters as well as the G-actin diffusion coefficient within reasonable ranges. We found that network growth with physiological parameter values always exceeds cellular dimensions. Filament severing [50] or filament burst disassembly [68] could further shorten the network. A treatment of these mechanisms is a reasonable extension of our model. As another mechanism, myosin-associated depolymerization by the convergence zone has been identified [27], which is implemented in our model. The physiological function of this structure likely goes beyond that of mere depolymerization. The convergence zone contracts the network, pulling the cell soma forward and contributing to the creation of retrograde flow [78]. Significant concentrations of filamentous actin in the back of the system are thus highly plausible since myosin motors need material to pull on in order to build a contractile apparatus. We believe that a deeper comprehension of this structure is an important next step towards a more faithful modeling and a deeper understanding of cell front organization.

While we can reproduce the characteristic filament length gradient at the front of the treadmilling array, our obtained total filament lengths in the back of the system (average ∼0.4 µm, maximum ∼1.5 µm; Figure 4B,C) do not match experimental data. Electron micrographs of the corresponding region in keratocytes show a predominance of filaments exceeding several micrometers in length [45], [79]. This discrepancy could be due to annealing and severing. Filament severing does not necessarily disassemble the network but could elicit additional polymerization [80] and counterintuitively even raise the mean filament length, due to rapid elongation of severed filaments which for a short time present free plus-ends to the highly concentrated actin monomer pool [12]. While these mechanisms' effects on filament lengths in the rear zone can be strong, the characteristic filament length gradient within the foremost 2 µm persists; the system's fluorescence signatures and the overall characteristics of the depolymerization source density profile are likewise conserved [12]. Our model results are hence meaningful despite neglecting severing and annealing due to their effect on only a subset of system features. Network architecture in cells is furthermore modulated by transient cross-linkers [81], [82]. For example, filamin is known to produce web-like actin networks [82] and might combine filaments in a way that no filament ends are discernible, as seen in electron micrographs [22], [45]. Likewise, formin proteins could mediate growth of long filaments from focal adhesions between lamellipodium and lamellum [70] or from the leading edge [71]. Since neither cross-linking mechanisms nor formin-induced filament elongation are included in our model, a match of absolute numbers of filament lengths cannot be expected.

We have assumed the same constant nucleation rate *N_0_* and hence the same plus-end concentration *B* and load per filament *f* in all calculations. As a consequence, the force-velocity relationship based on the thermal ratchet model (Equation 1) is effectively not probed in this work. Using a different force-velocity relationship would only rescale the network growth velocity and hence the spatial coordinate of all curves to the same extent. If, however, a nucleation rate dependence on, e.g., the leading edge G-actin or F-actin concentration was assumed [83], then the plus-end density and hence the force per filament would change with system parameters. The particular choice of the nucleation scenario and the force-velocity relationship would then leave its mesoscopic fingerprint in a differential scaling of individual curves in parameter studies such as in Figure S3. The same applies for a potential monomer concentration dependence of the force itself, as recently considered by Alt et al. [84].

Several of our model's predictions can be tested experimentally. In contrast to ADF/cofilin, tropomyosin signals have not yet been recorded in migrating keratocytes. A tropomyosin stain in keratocytes in accordance with the postulated tropomyosin signal (Figure 3) would support our hypothesized mechanism of competition between tropomyosin and ADF/cofilin, including our quantitative estimates for the binding kinetics. Second, a cell perturbation study blocking myosin motors in the convergence zone (e.g., with blebbistatin) could elucidate this zone's relative importance for network disassembly compared to filament severing and burst disassembly associated mechanisms as reported by Kueh et al. [68]. Cell perturbation experiments are valuable tools for examining treadmilling network mechanisms [24], [25], but for conclusive tests of our quantitative predictions, studies will have to more precisely control and systematically vary reaction kinetics. Systematic parameter studies could be better achieved using advanced biomimetic systems which for testing our model must allow tight control of the total actin content in flat geometries. The latter aspect is crucial to properly simulate the quasi-two-dimensional nature of diffusion in the lamellipodium/lamellum and will pose challenges to microfabrication.

With our model we contribute to a fundamental understanding of the formation of the lamellipodium and the lamellum, and of the concerted effects of network regulators such as tropomyosin and ADF/cofilin, as well as of the convergence zone on the decay and recycling of these networks. A comprehension of how cells organize their cytoskeletal components for achieving movement is an important goal within the life sciences, but is also of significant interest for medicine and materials science. If we grasp the physico-chemical mechanisms underlying the self-organized physiological functioning of cells, we may eventually harness this knowledge to manipulate intracellular processes to cure diseases, or build novel active biomimetic materials.

## Supporting Information

Figure S1
**Illustration of the calculation of F-actin concentration profiles as detailed in the** **Methods** **section**. Filaments with the same capped time *τ_c_* (“group *τ_c_*”, Figure 1) share a common plus-end position. Two exemplary filament groups are shown, 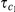
(left box) and 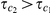 (right box). Left box: The contribution of the group 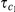 (plus-end position 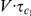) to the F-actin concentration at position *x_1_* (upper plot, left circular mark) corresponds to the number of filaments of this group crossing *x_1_*. The according condition 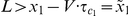 is fulfilled by all filaments uncapped longer than 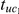 (lower left plot), where 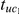
is calculated from Equation (9). The number of these filaments corresponds to the indicated surface under the *θ*(*t_uc_*) curve (lower right plot; Equation 8). To cross the more distant position 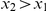, filaments must be longer and hence must have been uncapped longer (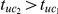), thus making up a smaller group fraction (decreased surface under the *θ*(*t_uc_*) curve corresponding to concentration value indicated by the right circular mark). Right box: Filaments of the group 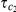 are consistently shorter than those of the group 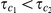, due to a longer duration of capping (upward shift of *t_uc_*(*L*) in the lower left plot compared to group 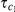 in the left box). Fewer filaments reach the exemplary probe lengths 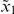 and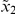. The F-actin concentration contribution of the group 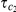 is thus lower than that of the group 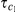 (compare upper plots in left and right box). The total F-actin concentration profile of the system is obtained by integration of all group contributions 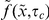 (Equations 11–13).(5.79 MB TIF)

Figure S2 **Model solutions under the assumption of constant minus end rate** ***r^−^*** **(** ***t*** **) = const.** This simplification allows semi-analytical solutions (solid lines; derivation in Text S3). Numerically calculated data (A, B: dashed lines; C: bars) in close agreement provide evidence for the validity of the facilitated numerical methods in this work.
(5.37 MB TIF)

Figure S3 **Regulatory effects of filament stabilizers and destabilizers.** Effects of tropomyosin and ADF/cofilin on network kinetics (rows 1–2), topology (row 3), and kinematics (row 4), as detailed in Text S4. All parameters are set to values given in Table 1 except for those under explicit variation (A–D: ADF/cofilin binding rate *r_ac_*, E–H: tropomyosin binding rate *r_tm_*).
(0.81 MB TIF)

Text S1 **Biomolecular parameter values.**
(0.03 MB PDF)

Text S2 **Estimation of the error introduced by the identification of the dwell time** ***t_dwell_*** **with the time since filament nucleation** ***t*** **.**
(0.09 MB PDF)

Text S3 **Semi-analytical solutions for constant filament minus-end rate.**
(0.03 MB PDF)

Text S4 **Regulatory effects of filament stabilizers and destabilizers.**
(0.01 MB PDF)
