# Supplementary figures and images for: Robust Organizational Principles of Protrusive Biopolymer Networks in Migrating Living Cells
Source: PLoS One. 2011 Jan 18;6(1):e14471. doi: 10.1371/journal.pone.0014471 (PMC3022574; doi:10.1371/journal.pone.0014471)

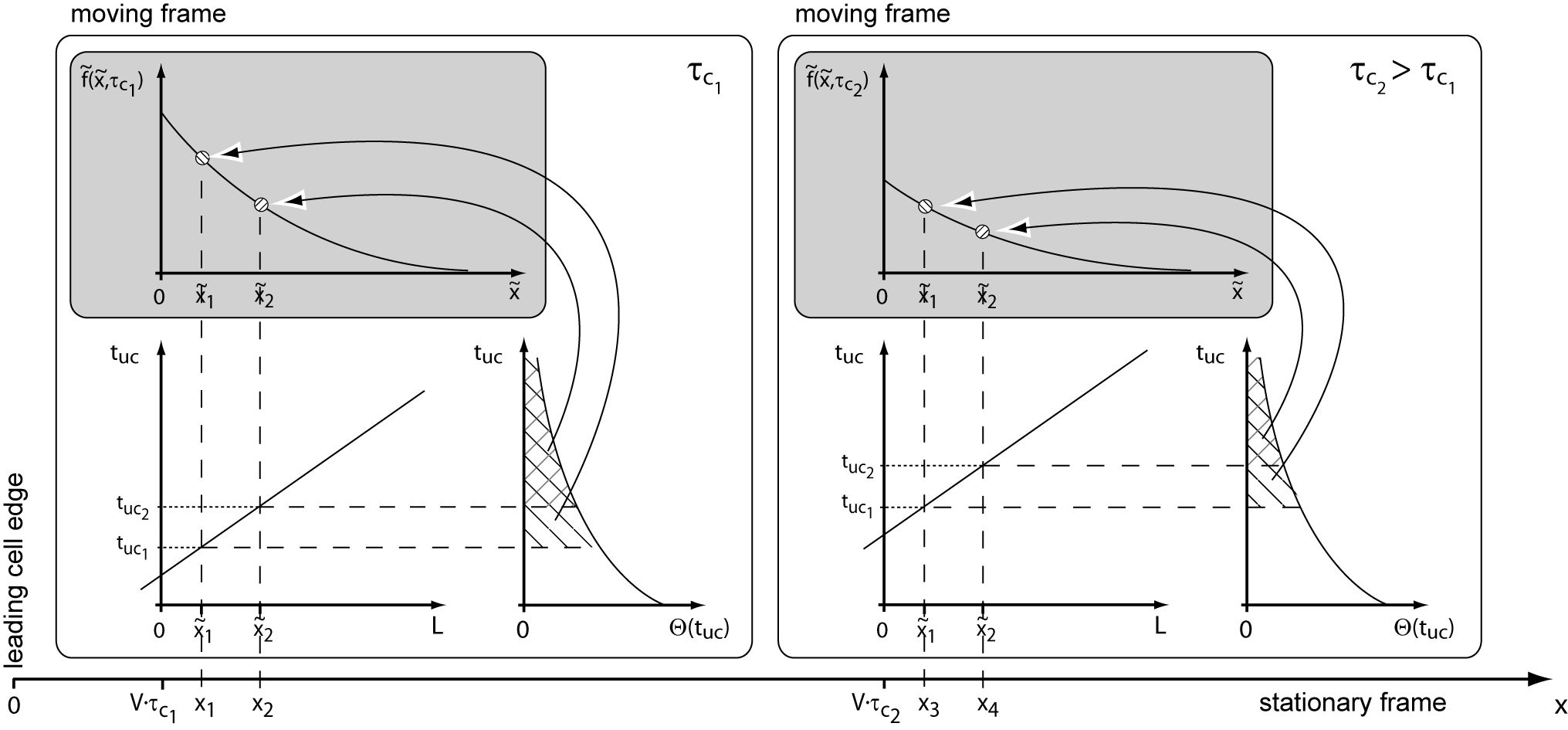

Supplement: Figure S1 — Illustration of the calculation of F-actin concentration profiles as detailed in the Methods section. Filaments with the same capped time τc (“group τc”, Figure 1) share a common plus-end position. Two exemplary filament groups are shown, (left box) and (right box). Left box: The contribution of the group (plus-end position ) to the F-actin concentration at position x1 (upper plot, left circular mark) corresponds to the number of filaments of this group crossing x1. The according condition is fulfilled by all filaments uncapped longer than (lower left plot), where is calculated from Equation (9). The number of these filaments corresponds to the indicated surface under the θ(tuc) curve (lower right plot; Equation 8). To cross the more distant position , filaments must be longer and hence must have been uncapped longer (), thus making up a smaller group fraction (decreased surface under the θ(tuc) curve corresponding to concentration value indicated by the right circular mark). Right box: Filaments of the group are consistently shorter than those of the group , due to a longer duration of capping (upward shift of tuc(L) in the lower left plot compared to group in the left box). Fewer filaments reach the exemplary probe lengths and. The F-actin concentration contribution of the group is thus lower than that of the group (compare upper plots in left and right box). The total F-actin concentration profile of the system is obtained by integration of all group contributions (Equations 11–13). (5.79 MB TIF) [file pone.0014471.s001.tif]

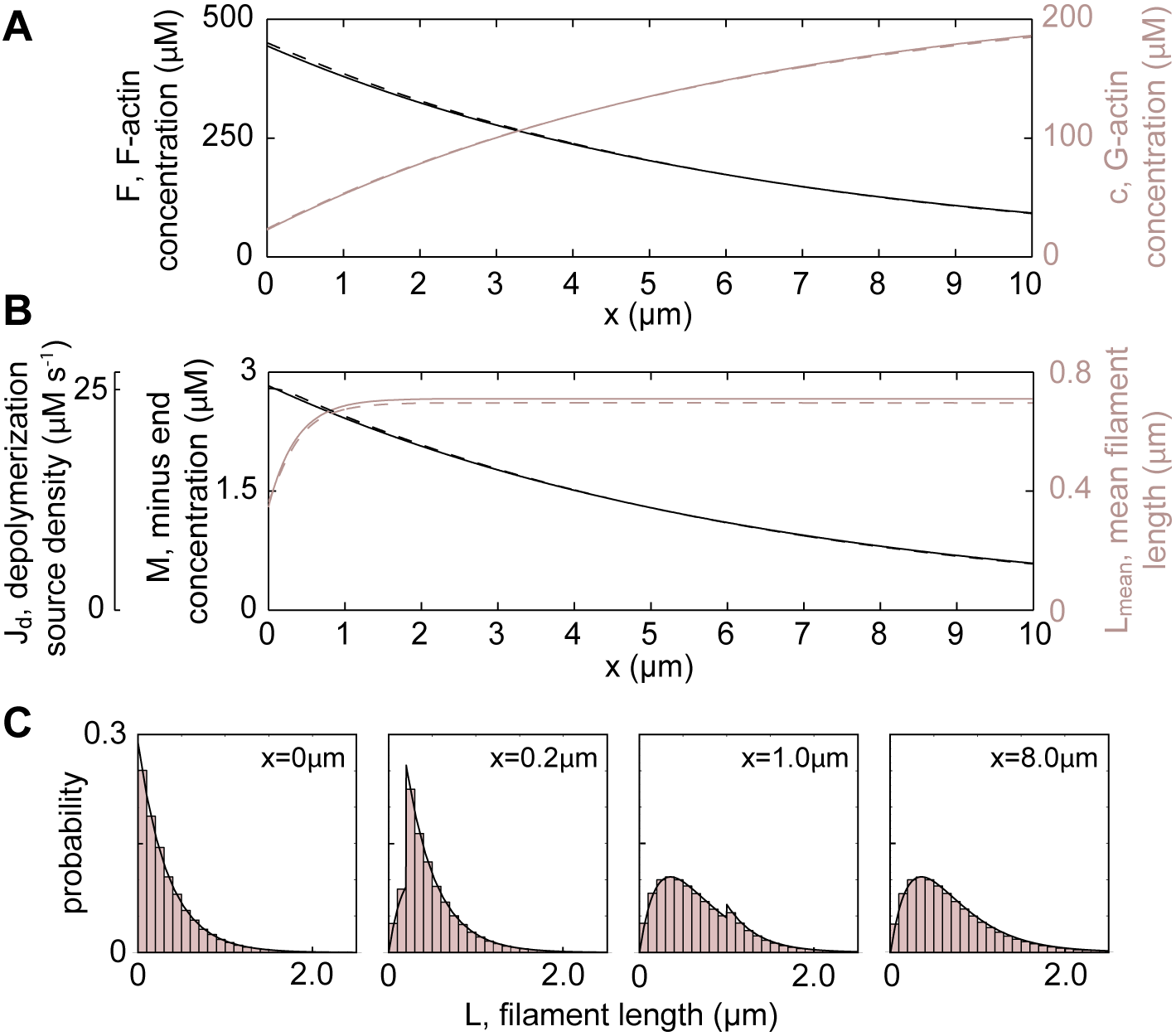

Supplement: Figure S2 — Model solutions under the assumption of constant minus end rate r− ( t ) = const. This simplification allows semi-analytical solutions (solid lines; derivation in Text S3). Numerically calculated data (A, B: dashed lines; C: bars) in close agreement provide evidence for the validity of the facilitated numerical methods in this work. (5.37 MB TIF) [file pone.0014471.s002.tif]

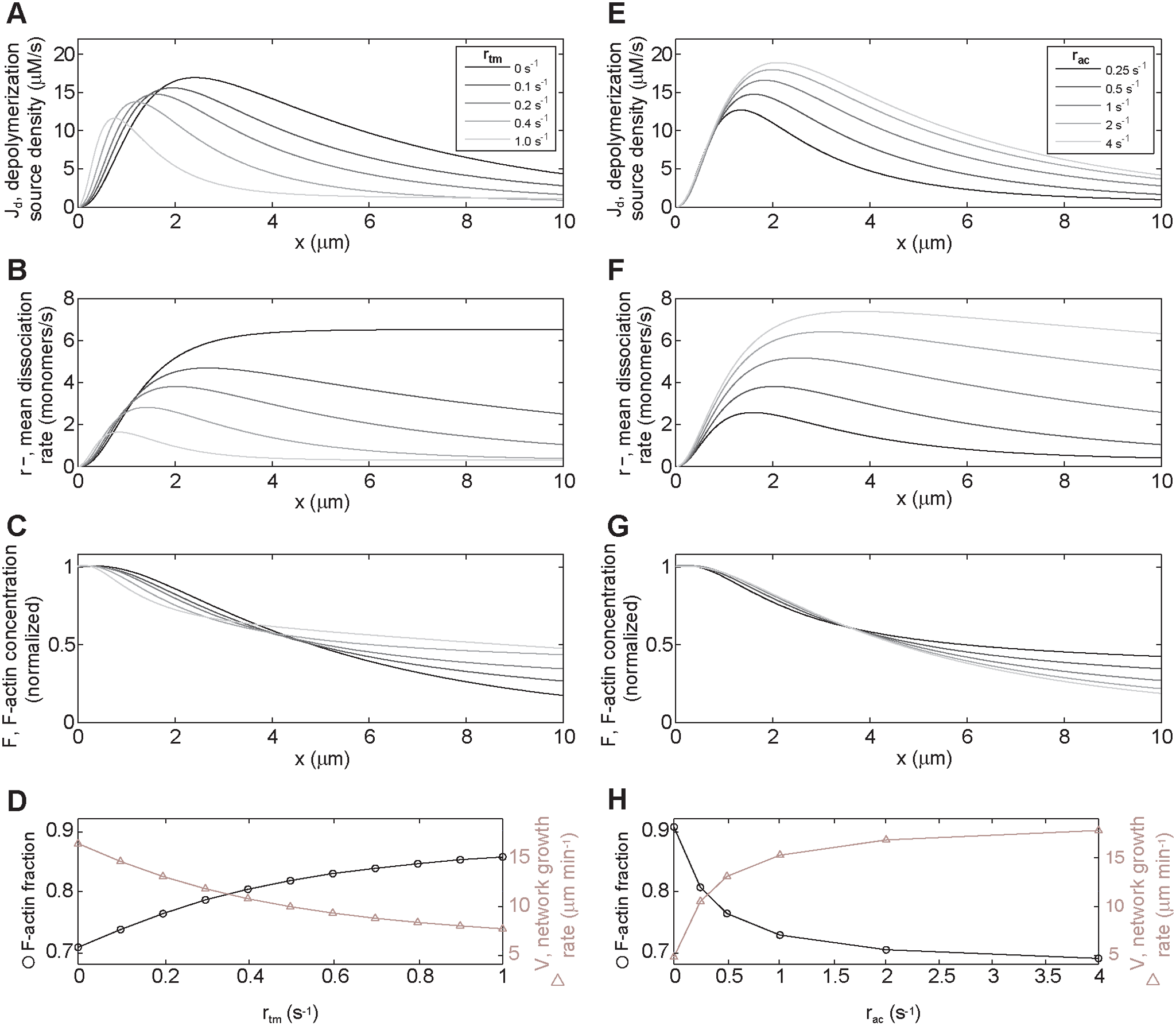

Supplement: Figure S3 — Regulatory effects of filament stabilizers and destabilizers. Effects of tropomyosin and ADF/cofilin on network kinetics (rows 1–2), topology (row 3), and kinematics (row 4), as detailed in Text S4. All parameters are set to values given in Table 1 except for those under explicit variation (A–D: ADF/cofilin binding rate rac, E–H: tropomyosin binding rate rtm). (0.81 MB TIF) [file pone.0014471.s003.tif]
