# Supplementary material for: Robust Organizational Principles of Protrusive Biopolymer Networks in Migrating Living Cells
Source: PLoS One. 2011 Jan 18;6(1):e14471. doi: 10.1371/journal.pone.0014471 (PMC3022574; doi:10.1371/journal.pone.0014471)
Supplement: Text S1 — Biomolecular parameter values. (0.03 MB PDF) [file pone.0014471.s004.pdf]

Text S1. Biomolecular parameter values

Total actin concentration

It is believed that most of the actin in the lamellipodium/lamellum is in its filamentous form. Various experimental methods have been used to measure actin concentrations in this cytoskeletal extension, yielding a broad range of results. Comparing coarse-grained simulation data with electron micrographs, actin concentrations of ~ 900 µM were found for keratocyte lamellipodia [1]. Fleischer et al. [2] obtained concentrations of ~ 250 µM by comparing tessellation models to electron micrographs. Huber et al. [3] found best fits of Monte Carlo simulation data on array treadmilling with fluorescence micrographs using actin concentrations of ~ 350 µM. We use the value of Huber et al. in all our calculations, also in order to compare the results of the mathematical model with those of the simulation.

Nucleation

We focus on Arp2/3-driven nucleation and assume a nucleation rate which is independent of the G- and the F-actin concentrations at the leading edge. The leading edge plus-end density *B* can be inferred from stall force measurements on keratocyte lamellipodia using atomic force microscopes [4, 5]. Assuming the validity of the thermal ratchet model of Mogilner et al. [6], these stall forces correspond to ~ 100 pushing filaments per micrometer of the leading edge, in agreement with alternative measurements [7, 8]. Assuming a height of the cytoskeletal extension of h ~ 170 nm [8] and a filament length increment in x direction per monomer of δ_p ~ 2.2 nm [9, 10], this corresponds to a plus-end density of B ~ 440 µM in the 2.2 nm deep volume abutting the cell membrane. The plus-end capping rate can be estimated to be r_{cap} ~ 1 s^{-1} [11]. With Equation (7), the resulting nucleation rate used in our calculations is thus $N_0 = B\, r_{cap}$ ~ 440 µM s^{-1}.

Actin hydrolysis

In vitro experiments have shown that after addition to the filament plus-end, ATP-F-actin subunits are soon (half time ~ 2s) irreversibly hydrolyzed to their ADP-Pi analogs [9, 12]. Subsequent phosphate dissociation is slower than hydrolysis by two orders of magnitude [13] but is accelerated at least 18-fold in the presence of ADF/cofilin [14]. In vivo, phosphate release is even more drastically enhanced by unknown factors. Taking typical network growth rates of ~ 0.25 µm/s into account, the visualized spatial offset between F-actin and F-actin bound ADF/cofilin at the leading edge of motile keratocytes (1-2 µm; [15]) can only be explained with half times of ~ 2-3 s for the complete conversion process from ATP- to ADP-F-actin. Based on these observations, we chose a hydrolysis rate of $r_{hyd} = 0.3\ s^{-1}$ for the complete conversion process.

Debranching

In our model, filament debranching corresponds to minus-end detachment from an Arp2/3 complex. Debranching rates measured in vitro [16] are far too low to explain lamellipodial network design obtained by electron microscopy [17]. It is widely believed that other factors such as ADF/cofilin or mechanical load accelerate debranching in vivo [16, 18]. Arp2/3 induced branches are limited to the foremost ~ 1 µm of the lamellipodium, which, taking into account typical network growth rates of ~ 0.25 µm/s, corresponds to debranching rates r_{deb} of ~ 0.5 s^{-1} used in our calculations, in agreement with speckle microscopy measurements implying rates of 0.1 - 0.5 s^{-1} [19].

Tropomyosin and ADF/cofilin binding

Typical intracellular ADF/cofilin concentrations are in the range of 20-100 µM [20]. From our calculations we find a fraction of ~ 85-95% of the ADF/cofilin pool to be bound to F-actin, implying free ADF/cofilin concentrations of ~ 1-15 µM. ADF/cofilin binding rate constants are in the range of ~ 0.03-0.05 $\mu M^{-1}\ s^{-1}$ assuming non-cooperative binding [14, 21]. Taken together, this yields plausible ADF/cofilin binding rates of r_{ac} ~ 0.03-1 s^{-1}. We use r_{ac}=0.5 s^{-1} following Huber et al. [3]. ADF/cofilin unbinding and tropomyosin binding rates in

cells are likewise not precisely determined. Huber et al. found best agreement of simulation data with experimental data for an ADF/cofilin unbinding rate of r_{ac}^{-} ~ 0.2 s^{-1} and a tropomyosin binding rate of r_{tm} ~ 0.2 s^{-1}. The latter complies to biochemical assay data indicating a half time of tropomyosin assembly with actin filaments of ~ 1 s [22]. We adopted these values from Huber et al. in our calculations. In reference to experiments by Ono and Ono [23], dissociation of tropomyosin from actin was assumed to negligible on the short timescales of network turnover (~ 1 min).

Diffusion

The diffusion coefficient for G-actin, which is considerably lower in cytoplasm than in aequous medium and depends on local cytoarchitecture [24], has been estimated based on fluorescence recovery after photobleaching (FRAP) measurements to be ~ 5-6 $\mu m^2 s^{-1}$ in the bulk actin cytoskeleton of endothelial cells [25]. Similar values have been obtained by Zicha et al. [26] for the lamellum/lamellipodium of fibroblasts. We assume a G-actin diffusion coefficient $D = 5\ \mu m^2 s^{-1}$ in our calculations.

Network dissolution

We consider the actin network to dissolve below an F-actin concentration of 5 µM. This cutoff value defining the network length in the treatment of unconfined treadmilling (Results section "Unconfined treadmilling") is estimated by comparing the mean filament length with the average distance between filament cross-linking points in the network. Mean filament lengths are on the order of 0.5 µm (Figure 4B). The average distance between cross-linking points (in µm) for densely cross-linked networks is approximated by the mesh size $\xi \approx F^{-0.5}$, with F the F-actin concentration in $\mu m/\mu m^3$. Network dissolution will clearly occur where ξ exceeds the mean filament length, $\xi > 0.5$ µm, which corresponds to F-actin concentrations of several µM.

References

1. Schaub S, Meister J, Verkhovsky A (2007) Analysis of actin filament network organization in lamellipodia by comparing experimental and simulated images. J Cell Sci 120: 1491-1500.

2. Fleischer F, Ananthakrishnan R, Eckel S, Schmidt H, Käs J, et al. (2007) Actin network architecture and elasticity in lamellipodia of melanoma cells. New J Phys 9: 420.

3. Huber F, Käs J, Stuhrmann B (2008) Growing actin networks form lamellipodium and lamellum by self-assembly. Biophys J 95: 5508-5523.

4. Brunner C, Ehrlicher A, Kohlstrunk B, Knebel D, Käs J, et al. (2006) Cell migration through small gaps. Eur Biophys J 35: 713-719.

5. Prass M, Jacobson K, Mogilner A, Radmacher M (2006) Direct measurement of the lamellipodial protrusive force in a migrating cell. J Cell Biol 174: 767-772.

6. Mogilner A, Oster G (1996) Cell motility driven by actin polymerization. Biophys J 71: 3030-3045.

7. Koestler S, Auinger S, Vinzenz M, Rottner K, Small J (2008) Differentially oriented populations of actin filaments generated in lamellipodia collaborate in pushing and pausing at the cell front. Nat Cell Biol 10: 306-313.

8. Abraham VC, Krishnamurthi V, Taylor L, Lanni F (1999) The actin-based nanomachine at the leading edge of migrating cells. Biophys J 77: 1721-1732.

9. Pollard T, Borisy G (2003) Cellular motility driven by assembly and disassembly of actin filaments. Cell 112: 453-465.

10. Schaus T, Taylor E, Borisy G (2007) Self-organization of actin filament orientation in the dendritic-nucleation/array-treadmilling model. Proc Natl Acad Sci USA 104: 7086-7091.

11. Schafer D, Jennings P, Cooper J (1996) Dynamics of capping protein and actin assembly in vitro: uncapping barbed ends by polyphosphoinositides. J Cell Biol 135: 169-179.

12. Blanchoin L, Pollard T (2002) Hydrolysis of ATP by polymerized actin depends on the bound divalent cation but not profilin. Biochem 41: 597-602.

13. Melki R, Fievez S, Carlier M (1996) Continuous monitoring of Pi release following nucleotide hydrolysis in actin or tubulin assembly using 2-amino-6-mercapto-7-methylpurine ribonucleoside and purine-nucleoside phosphorylase as an enzyme-linked assay. Biochem 35: 12038-12045.

14. Blanchoin L, Pollard TD (1999) Mechanism of Interaction of Acanthamoeba Actophorin (ADF/Cofilin) with Actin Filaments. J Biol Chem 274: 15538-15546.

15. Svitkina T, Borisy G (1999) Arp2/3 complex and actin depolymerization factor/cofilin in dendritic organization and treadmilling of actin filament array in lamellipodia. J Cell Biol

145: 1009-1026.

16. Martin A, Welch M, Drubin D (2006) Arp2/3 ATP hydrolysis-catalysed branch dissociation is critical for endocytic force generation. Nat Cell Biol 8: 826-833.

17. Svitkina T, Verkhovsky A, McQuade K, Borisy G (1997) Analysis of the actin-myosin II system in fish epidermal keratocytes: mechanism of cell body translocation. J Cell Biol 139: 397-415.

18. Blanchoin L, Pollard T, Mullins R (2000) Interactions of ADF/cofilin, Arp2/3 complex, capping protein and profilin in remodeling of branched actin filament networks. Curr Biol 10: 1273-1282.

19. Watanabe N, Mitchison T (2002) Single-molecule speckle analysis of actin filament turnover in lamellipodia. Science 295: 1083-1086.

20. Pollard T, Blanchoin L, Mullins R (2000) Molecular mechanisms controlling actin filament dynamics in nonmuscle cells. Annu Rev Biophys Biomol Struct 29: 545-576.

21. Muhlrad A, Pavlov D, Peyser M, Reisler E (2006) Inorganic Phosphate Regulates the Binding of Cofilin to Actin Filaments. FEBS J 273: 1488-1496.

22. Weigt C, Wegner A, Koch M (1991) Rate and mechanism of the assembly of tropomyosin with actin filaments. Biochem 30: 10700-10707.

23. Ono S, Ono K (2002) Tropomyosin inhibits ADF/cofilin-dependent actin filament dynamics. J Cell Biol 156: 1065-1076.

24. Luby-Phelps K (2000) Cytoarchitecture and physical properties of cytoplasm: volume, viscosity, diffusion, intracellular surface area. Intl Rev Cytol 192: 189-221.

25. McGrath J, Tardy Y, Dewey C, Meister J, Hartwig J (1998) Simultaneous measurements of actin filament turnover, filament fraction, and monomer diffusion in endothelial cells. Biophys J 75: 2070-2078.

26. Zicha D, Dobbie I, Holt M, Monypenny J, Soong D, et al. (2003) Rapid actin transport during cell protrusion. Science 300: 142-145.
