# Supplementary material for: Robust Organizational Principles of Protrusive Biopolymer Networks in Migrating Living Cells
Source: PLoS One. 2011 Jan 18;6(1):e14471. doi: 10.1371/journal.pone.0014471 (PMC3022574; doi:10.1371/journal.pone.0014471)
Supplement: Text S2 — Estimation of the error introduced by the identification of the dwell time tdwell with the time since filament nucleation t . (0.09 MB PDF) [file pone.0014471.s005.pdf]

Text S2: Estimation of the error introduced by the identification of the dwell time t_{dwell} with the time since filament nucleation t

The dwell time of the terminal subunit of a filament t_{dwell}, i.e. the time the terminal subunit has spent in the filament, generally differs from the time since filament nucleation, t. As long as a filament is attached with its minus-end to a mother filament, $t_{dwell} = t$, but after debranching of a filament and the beginning of its depolymerization, $t_{dwell} < t$, where the difference between t_{dwell} and t depends on the time since debranching. In contrast, Equation (4) assumes $t_{dwell} = t$ for all filaments, to prevent highly nonlinear equations which were impossible to solve. The maximum error introduced by this approximation is estimated as follows. The maximum deviation between t_{dwell} and t in a filament certainly occurs in the case of instantaneous debranching and instantaneous binding of ADF/cofilin. The minus-end rate of a filament is then constant and reads $s_{ac}\, k_{off}^{-}$. The position of the filament's minus-end, $x_m = V\, t_{dwell}$, is in this case described as $x_m = V t - \delta_p\, s_{ac}\, k_{off}^{-}\, t$, and hence

$$\frac{t}{t_{dwell}} = \frac{1}{1 - \frac{\delta_p\, s_{ac}\, k_{off}^{-}}{V}}. \qquad \text{(S1.1)}$$

For typical network rates of V = 10 µm/min, t/t_{dwell} =1.14. Equation (4) averages over all filaments of the system; by assuming $t_{dwell} = t$ in Equation (4), the arguments used for the state probabilities p_{adp}, p_{tm}, and p_{ac} exceed any individual filament's actual t_{dwell}. Equation (S1.1) now gives an upper bound of this overestimation; no individual filament will ever experience a larger discrepancy between t and t_{dwell}. We can recalculate the minus-end rate with the state probabilities' arguments rescaled by $t_{dwell}/t = 1.14^{-1} = 0.88$, and compare the model solution based on this new minus-end rate with the original one. The difference between these two solutions represents an upper bound for the error introduced by assuming $t_{dwell} = t$.

We found the two solutions to agree reasonably well (see figure; mean relative deviations along curves: F-actin concentration 2.3%, ADF/cofilin-F-actin concentration 13%, tropomyosin-F-actin concentration 1.9%, depolymerization source density 9.7%, mean filament length 0.9%). In particular, the distinctive molecular, kinetic, and structural system characteristics are conserved and vary only little in position; most notable are shifts of the maximum of ADF/cofilin-F-actin signal and of the depolymerization source density 0.3 µm away from the leading edge. The approximation $t_{dwell} = t$ thus seems reasonable.

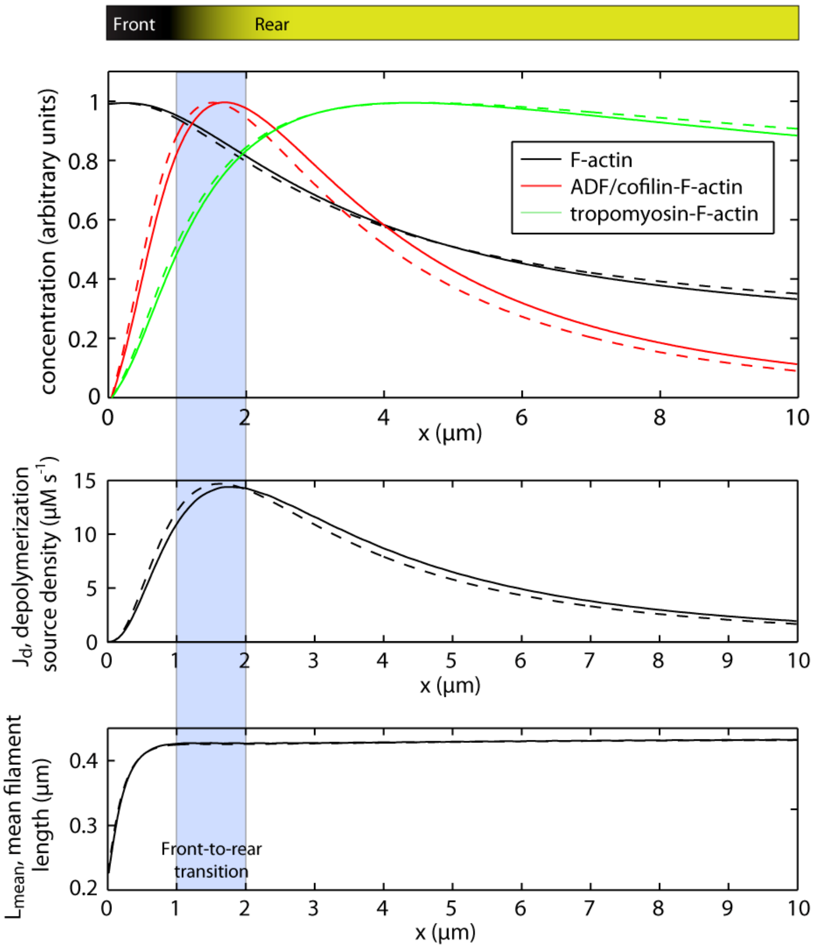

Figure: Model solutions with the state probabilities' arguments in Equation (4) rescaled by $t_{dwell}/t = 0.88$ (solid line) and original solutions assuming $t_{dwell} = t$ for all filaments (dashed lines), illustrating the maximum error introduced by the approximation $t_{dwell} = t$.
