# Supplementary material for: Robust Organizational Principles of Protrusive Biopolymer Networks in Migrating Living Cells
Source: PLoS One. 2011 Jan 18;6(1):e14471. doi: 10.1371/journal.pone.0014471 (PMC3022574; doi:10.1371/journal.pone.0014471)
Supplement: Text S3 — Semi-analytical solutions for constant filament minus-end rate. (0.03 MB PDF) [file pone.0014471.s006.pdf]

Text S3. Semi-analytical solutions for constant filament minus-end rate

Closed analytical expressions for the set of coupled equations derived in the Methods section can be found with the following simplifying assumptions: a) Instant binding of ADF/cofilin to a subunit after its incorporation into a filament. b) Absence (or alternatively immediate binding) of tropomyosin. c) Immediate filament debranching after nucleation. The minus-end depolymerization rate in this particular scenario is constant and reads $r^- = s_{ac}\, k_{off}^-$. Hence $L(t_{uc}, \tau_c) = \delta_p \left(r^+ t_{uc} - s_{ac}\, k_{off}^- (\tau_c + t_{uc}) \right)$, and the lower boundary for the integration over filaments within a group becomes $t_{uc} = (\tilde{x} + (V - V_{rel})\,\tau_c)/V_{rel}$ with $V \equiv \delta_p\, r^+$ the network growth rate and $V_{rel} \equiv \delta_p\,(r^+ - s_{ac}\, k_{off}^-)$ the relative velocity of the endpoints of a filament. Evaluation of Equations (11)-(13) with these integration limits yields the F-actin concentration profile

$$F(x) = B \exp(-q\, x) \tag{S3.1}$$

with $q \equiv r_{cap}\,(V - V_{rel})/(V\, V_{rel})$. The fraction of filaments of the group τ_c with length *L* exceeding $\tilde{x}$ is (Equation 14) $\tilde{p}(\tau_c; L, \tilde{x})\, dL = r_{cap}/V_{rel} \exp(-r_{cap}\,(L - \tilde{x})/V_{rel})\, \mathrm{H}(L - \tilde{x})\, dL$, which with Equation (15) yields

$$P(L, x) = \frac{r_{cap}}{V - V_{rel}} \exp\left(-\frac{r_{cap}}{V_{rel}} L\right) \left\{ \mathrm{H}(x - L)\left[\exp(q\, L) - 1\right] + \mathrm{H}(L - x)\left[\frac{V}{V_{rel}} \exp(q\, x) - 1\right] \right\} \tag{S3.2}$$

as the probability (in μm^{-1}) that a filament transecting position *x* has a length *L*. Equation (16) cannot be solved analytically using this expression. A reversal of the order of

integration resolves this problem: integration of all possible values of L, weighted by the coefficients $\tilde{p}(\tau_c; L, \tilde{x})$, yields the mean length of filaments of the group τ_c at distance $\tilde{x}$ from the group's plus-ends, $\tilde{L}_{mean}^{group}(\tau_c, \tilde{x}) = \int_0^\infty L\ \tilde{p}(\tau_c; L, \tilde{x})\, dL$. Transformation into the stationary frame, $L_{mean}^{group}(\tau_c, x) = \mathrm{H}(x - V\tau_c)\ \tilde{L}_{mean}^{group}(\tau_c, x - V\tau_c)$, and integration of all group contributions weighted by each group's share of the total F-actin concentration at position x, $\frac{f(x,\tau_c)\, d\tau_c}{F(x)}$, yields the mean filament length profile $L_{mean}(x) = \int_0^\infty L_{mean}^{group}(\tau_c, x) \frac{f(x,\tau_c)}{F(x)} d\tau_c + L_{mean}^{group}(0, x) \frac{F_{uc}(x)}{F(x)}$, which for the particular scenario discussed here is

$$L_{mean}(x) = r_{cap}^{-1} \left(V\,(1 - \exp(-r_{cap} \frac{x}{V})) + V_{rel} \right). \qquad \text{(S3.3)}$$

The groups' minus-end concentration contributions read $\tilde{m}(\tilde{x}, \tau_c)\, d\tau_c = -\delta_p\, (\partial / \partial \tilde{x})\ \tilde{f}(\tilde{x}, \tau_c)\, d\tau_c = \delta_p\, r_{cap} / V_{rel}\ \tilde{f}(\tilde{x}, \tau_c)\, d\tau_c$, which after transformation into the stationary frame and integration over all groups yields the minus-end concentration profile

$$M(x) = \frac{\delta_p\, r_{cap}}{V_{rel}} F(x) \qquad \text{(S3.4)}$$

and the depolymerization source density

$$J_d(x) = \frac{V - V_{rel}}{V_{rel}} r_{cap}\, F(x). \qquad \text{(S3.5)}$$

By solving the equation $(\partial / \partial x)^2 c = -J_d(x) / D$ using boundary conditions discussed in the text (Equations 20 and 21), the G-actin concentration profile *c(x)* is obtained as

$$c(x) = \frac{1}{L_{sys}\, q}\left(\frac{BV}{D\,q} - B\right)(1 - \exp(-q\, L_{sys})) - \frac{BV}{D\,q}\exp(-q\, x) + A\,, \qquad \text{(S3.6)}$$

where *A* is the average total actin concentration in the system.

The G-actin concentration at the leading edge *c*(*0*) is obtained by solving Equation (S3.6) for *c*(*0*). This can only be done numerically. A minimum of numerical calculation thus remains even with this simplified set of equations describing network treadmilling.
