# Supplementary material for: Robust Organizational Principles of Protrusive Biopolymer Networks in Migrating Living Cells
Source: PLoS One. 2011 Jan 18;6(1):e14471. doi: 10.1371/journal.pone.0014471 (PMC3022574; doi:10.1371/journal.pone.0014471)
Supplement: Text S4 — Regulatory effects of filament stabilizers and destabilizers. (0.01 MB PDF) [file pone.0014471.s007.pdf]

Text S4. Regulatory effects of filament stabilizers and destabilizers

The regulatory effect of any of the specific actin binding proteins in our system can best be understood by variation of this protein's binding rate (i.e. of its intracellular concentration).

In our model, binding kinetics of the filament stabilizer tropomyosin sensitively affect network degradation. Without tropomyosin, depolymerization is prominent within 2-3 µm from the leading edge (Figure S3A). Upon raising the tropomyosin binding rate, depolymerization is attenuated and becomes much more pronounced in the foremost region: the depolymerization source density profile constricts, and its peak moves as close as 1 µm to the leading edge, which we interpret as a decrease of lamellipodium size. This change in depolymerization source density is in part the result of a decreased network growth rate (7.6 µm/min for r_{tm}=1.0 s^{-1} as opposed to 16 µm/min without tropomyosin; Figure S3D) due to the majority of actin subunits being bound in a tropomyosin-stabilized network. It also results from a drop in the mean dissociation rate towards the back, reflecting tropomyosin's competition with ADF/cofilin for actin binding (Figure S3B). The alterations in depolymerization and network growth speed influence the F-actin concentration such that with tropomyosin, the F-actin profile declines more steeply at the front but then levels off at higher values than in the tropomyosin-free case (Figure S3C), while the fraction of filamentous actin in the system increases (Figure S3D). We identify this trend as stabilization of the network.

The regulative effects of ADF/cofilin can be understood in a similar manner. Upon raising the ADF/cofilin binding rate, the mean dissociation rate (Figure S3F) and consequently the depolymerization source density (Figure S3E) increase, concomitant with a shift of the depolymerization maximum towards the back (Figure S3E). This shift is mainly

due to an increase of network growth rate (Figure S3H), which results from the enhanced supply of the leading edge with G-actin monomers freed from the network by the action of ADF/cofilin. Ultimately, the altered network kinetics result in a steeper decline of the F-actin profile in the presence of ADF/cofilin - despite the enhanced network growth rate - compared to the ADF/cofilin-free case (Figure S3G), and in a reduced F-actin fraction (Figure S3H). In this sense, ADF/cofilin destabilizes the network.

The calculated decrease in lamellipodium width with increasing tropomyosin levels (Figure S3A) is in agreement with tropomyosin microinjection [1] and gene silencing [2] studies. Enhanced network growth velocities as observed by Gupton et al. [1], however, cannot be reproduced and are likely related to myosin-induced contraction, which is out of the scope of our model. An acceleration of network turnover with enhanced ADF/cofilin activity is in agreement with findings from cell perturbation experiments showing that network growth rates [2-4] (compare Figure S3H) and depolymerization [3] (compare Figure S3E) increase with increasing ADF/cofilin activity. The calculated trend of a decreasing F-actin fraction (Figure S3H) likewise reproduces experimental data [2, 5, 6], and the ADF/cofilin-induced shift of the depolymerization source density maximum away from the leading edge (Figure S3E) complies with a widening of the lamellipodium observed by Delorme et al. [3].

References

1. Gupton S, Anderson K, Kole T, Fischer R, Ponti A, et al. (2005) Cell migration without a lamellipodium: translation of actin dynamics into cell movement mediated by tropomyosin. J Cell Biol 168: 619-631.

2. Iwasa J, Mullins R (2007) Spatial and temporal relationships between actin-filament nucleation, capping, and disassembly. Curr Biol 17: 395-406.

3. Delorme V, Machacek M, DerMardirossian C, Anderson K, Wittmann T, et al. (2007) Cofilin activity downstream of Pak1 regulates cell protrusion efficiency by organizing lamellipodium and lamella actin networks. Dev Cell 13: 646-662.

4. Ghosh M, Song X, Mouneimne G, Sidani M, Lawrence D, et al. (2004) Cofilin promotes actin polymerization and defines the direction of cell motility. Science 304: 743-746.

5. Hotulainen P, Paunola E, Vartiainen M, Lappalainen P (2005) Actin-depolymerizing factor and cofilin-1 play overlapping roles in promoting rapid F-actin depolymerization in mammalian nonmuscle cells. Mol Biol Cell 16: 649-664.

6. Kiuchi T, Ohashi K, Kurita S, Mizuno K (2007) Cofilin promotes stimulus-induced lamellipodium formation by generating an abundant supply of actin monomers. J Cell Biol 177: 465-476.
